# Highly‐Selective Harvesting of (6,4) SWCNTs Using the Aqueous Two‐Phase Extraction Method and Nonionic Surfactants

**DOI:** 10.1002/advs.202207218

**Published:** 2023-03-01

**Authors:** Blazej Podlesny, Kevin R. Hinkle, Keita Hayashi, Yoshiaki Niidome, Tomohiro Shiraki, Dawid Janas

**Affiliations:** ^1^ Department of Organic Chemistry Bioorganic Chemistry and Biotechnology Silesian University of Technology B. Krzywoustego 4 Gliwice 44‐100 Poland; ^2^ Department of Chemical and Materials Engineering University of Dayton Dayton OH 45469 USA; ^3^ Department of Applied Chemistry Graduate School of Engineering Kyushu University 744 Motooka, Nishi‐ku Fukuoka 819‐0395 Japan

**Keywords:** interactions, purification, single‐walled carbon nanotubes, surfactants

## Abstract

Monochiral single‐walled carbon nanotubes (SWCNTs) are indispensable for advancing the technology readiness level of nanocarbon‐based concepts. In recent times, many separation techniques have been developed to obtain specific SWCNTs from raw unsorted materials to catalyze the development in this area. This work presents how the aqueous two‐phase extraction (ATPE) method can be enhanced for the straightforward isolation of (6,4) SWCNTs in one step. Introducing nonionic surfactant into the typically employed mixture of anionic surfactants, which drive the partitioning, is essential to increasing the ATPE system's resolution. A thorough analysis of the parameter space by experiments and modeling reveals the underlying interactions between SWCNTs, surfactants, and phase‐forming agents, which drive the partitioning. Based on new insight gained on this front, a separation mechanism is proposed. Notably, the developed method is highly robust, which is proven by isolating (6,4) SWCNTs from several raw SWCNT materials, including SWCNT waste generated over the years in the laboratory.

## Graphical abstract

Reaching structural homogeneity in nanomaterials is crucial for drawing meaningful conclusions about their nature. This contribution enhances the resolution and elucidates the mechanism of the aqueous two‐phase extraction method, which has considerable application potential for purifying many classes of nanomaterials. The application of unrecognized combination of surfactants leads to highly selective one‐step separation of (6,4) single‐walled carbon nanotubes.

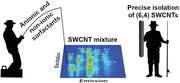

## 1 Introduction

Single‐walled carbon nanotubes (SWCNTs) exhibit dramatically different characteristics depending on the arrangement of carbon atoms in a nanotube.^[^ ^1^ ^]^ Consequently, the proper structure is required to make the particular SWCNT suitable for a specific application. When this condition is fulfilled, implementation opportunities of SWCNTs broaden very much. For instance, SWCNTs of a defined morphology constitute a significant asset in medicine (e.g., theranostic agents^[^ ^2^ ^]^), optoelectronics^[^ ^3^ ^]^ (e.g., light emitters), and electronics^[^ ^4^ ^]^ (e.g., transistors). However, the field still lacks effective, scalable, and low‐cost methods of SWCNTs synthesis with specific structures.^[^ ^5^ ^]^

Thus, to reach this goal, obtained polydisperse SWCNT mixtures must be sorted.^[^ ^5^, ^6^ ^]^ Over the last decade, a spectrum of sorting methods has been developed for this purpose, including chromatography,^[^ ^7^ ^]^ electrophoresis,^[^ ^8^ ^]^ ultracentrifugation,^[^ ^9^ ^]^ etc. Among the available tools to differentiate SWCNTs, the aqueous two‐phase extraction (ATPE) method appears especially appealing due to its multiple merits.^[^ ^10^ ^]^ ATPE is a type of liquid‐liquid extraction in which both liquid phases are aqueous solutions of sufficiently different properties to enable phase separation.^[^ ^11^ ^]^ A two‐phase aqueous system is typically induced using concentrated solutions of two polymers^[^ ^12^ ^]^ or a combination of polymer and salt.^[^ ^13^ ^]^ In the case of SWCNT separation, usually, a polymer–polymer system consisting of dextran (DEX) and polyethylene glycol (PEG) is used^[^ ^10^ ^]^ (less commonly PEG/polyacrylamide^[^ ^14^ ^]^ or polyvinylpyrrolidone/DEX^[^ ^14^ ^]^). The extraction is driven by introducing surfactants^[^ ^15^ ^]^ and other modulators (e.g., basic/acidic compounds^[^ ^16^ ^]^ or redox agents^[^ ^17^ ^]^), which change how SWCNTs are partitioned between the phases. The selection of appropriate extraction conditions gives SWCNTs of particular diameter,^[^ ^18^ ^]^ electronic type,^[^ ^19^ ^]^ or even handedness.^[^ ^20^ ^]^ Despite these breakthroughs, the separation mechanism is still not fully understood, so the extraction routines often require several steps to isolate specific SWCNTs.

Surfactants exhibit strong interactions with a plethora of compounds and materials, so they are commonly used in the ATPE process to modify its course. Once they deposit on the SWCNT surface, the physicochemical nature of SWCNTs changes substantially, enabling this material's purification by the ATPE method. The current understanding is that if the surfactant is predominantly hydrophobic, the SWCNTs encapsulated by its molecules will migrate to the more hydrophobic phase. This effect has been demonstrated multiple times using the DEX‐PEG (bottom‐top) partitioning system, wherein sodium dodecyl sulfate (SDS), as well as sodium dodecylbenzene sulfonate (SDBS) surfactants, promoted the upward shift of SWCNTs.^[^ ^21^ ^]^ That is because SDS or SDBS are more hydrophobic, and SWCNTs coated by SDS or SDBS migrate to the more hydrophobic (PEG‐rich) top phase. Conversely, sodium deoxycholate (DOC) or sodium cholate (SC) are more hydrophilic, which favors the shift of SWCNTs to the more hydrophilic (DEX‐rich) bottom phase. Application of this relation enabled the stepwise separation of individual species from the raw materials, both for small^[^ ^22^ ^]^ and large^[^ ^23^ ^]^ diameter SWCNTs, by tuning the ATPE system to reach the appropriate amounts of SDS/SDBS and DOC/SC, thus enabling differentiation of SWCNTs between phases.

The surfactant selection also significantly impacts the purification of SWCNTs when other than ATPE separation methods are used. For example, in the case of column chromatography, it was observed that the SDS solution elutes only the weakly adsorbed metallic species from the agarose stationary phase. In contrast, DOC solution allows desorption and separation of the semiconducting species too.^[^ ^24^ ^]^ Also, SWCNT fractionation by density gradient ultracentrifugation is strongly affected by the type of surfactant employed. For instance, the application of SDBS as an SWCNT dispersant makes the isolation ineffective, causing the material to agglomerate into a single band. At the same time, the alternative use of SC enables the differentiation of the starting SWCNT material.^[^ ^9^ ^]^

It can be concluded that the main‐stream use of surfactants for the separation of SWCNTs has been limited to four anionic compounds: DOC,^[^ ^20^ ^]^ SC,^[^ ^21^ ^]^ SDS,^[^ ^23^ ^]^ and SDBS.^[^ ^25^ ^]^ Still, many other surfactants (both ionic and nonionic) could be exploited in the ATPE. To fill this niche, the separation capabilities of the ATPE method have been recently expanded to include other bile salts^[^ ^26^ ^]^ and a nonionic surfactant.^[^ ^15^ ^]^ However, this research is only in its infancy, so the knowledge of the underlying interactions between these surfactants and SWCNTs is lacking.

In this work, we present how nonionic surfactants can dramatically enhance the resolution of the ATPE system. Analysis of the ATPE system facilitated by various nonionic surfactants at different concentrations enabled us to tailor the extraction parameters to isolate pure fractions of (6,4)‐SWCNT chirality in one step (even from raw materials poor in (6,4) SWCNTs or waste SWCNT materials accumulated over the years). Furthermore, a thorough investigation of the underlying surfactant‐SWCNT interactions by molecular modeling explained the modus operandi of the ATPE system promoted by nonionic surfactants. Interestingly, as the results of this study show, the successful separation can already be programmed at the point of preparation of SWCNT suspension for subsequent sorting. Consequently, this report paves the way to the facile extraction of (6,4) SWCNTs at a large scale, thereby broadening application opportunities for small‐diameter species, which are of high practical utility.

## 2 Results and Discussion

### 2.1 Analysis of Starting Material

**Figure 1:**
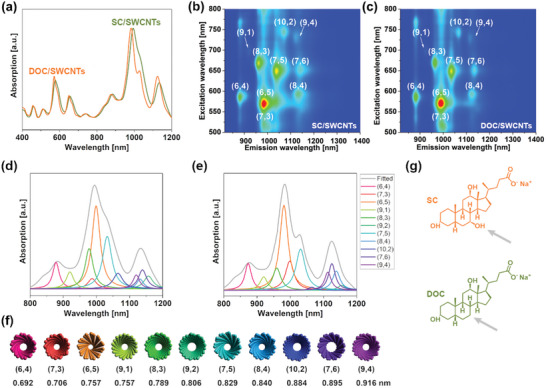
Characterization of the starting material dispersed in SC or DOC. a) Absorption spectra. 2D PLE maps of SG65i dispersion in b) SC and c) DOC. Individual optical transitions of the detected SWCNT species for d) SC and e) DOC SWCNT dispersion, respectively, were deconvoluted using PTF Fit application.^[^ ^27^ ^]^ The background, modeled by the function reported by Nair et al. was subtracted, and individual components were resolved using Voigt lineshapes.^[^ ^28^ ^]^ f) Specific types of detected SWCNT types (not to scale). g) The difference in chemical structures between SC and DOC.

For the majority of our experiments, we selected (6,5)‐enriched CoMoCAT SWCNTs (called SG65i in text). We prepared two starting dispersions in SC and DOC, both in 2% concentration of surfactant and 1 mg mL^−1^ SWCNT concentration (**Figure** **1**a).

Although both absorption spectra had a similar shape, with the domination of peaks from (6,5) chirality, we noticed better SWCNTs individualization for DOC dispersion as this surfactant has a stronger affinity to SWCNTs.^[^ ^29^ ^]^ In addition, more complete coverage of SWCNTs with DOC changed the dielectric environment around the SWCNTs,^[^ ^30^ ^]^ manifested herein by a slight hypsochromic shift to the absorption spectrum.^[^ ^31^ ^]^ As expected, other species than (6,5) SWCNTs were also detected in both suspensions. Regardless of the bile salts used, more than ten semiconducting species were detected (Figure 1b,c). Due to the unalike affinity of these two surfactants to SWCNTs, the 2D PL maps, as well as deconvoluted absorption spectra,^[^ ^27^ ^]^ revealed a slightly dissimilar abundance of individual species (Figure 1d–f), even though these two surfactants differ only by one hydroxyl group (Figure 1g). Because these surfactants are commonly used interchangeably by the scientific community, this variation should be taken into account.

### 2.2 Partitioning Using DOC SWCNT Dispersion and DOC/TX100 Surfactant Mixture

In principle, the outcome of ATPE separation results from competition between the surfactants present in the system. Depending on the structure, surfactants are more or less hydrophilic/hydrophobic, which translates directly into the migration of surfactant‐nanotube hybrids to the preferred phases. Phase‐forming polymers in the ATPE system also differ significantly in polarity, which, based on the structure of the surfactant, allows for predicting the migration preferences of the SWCNT‐surfactant hybrid to the extraction phases. As indicated above, in a PEG‐dextran ATPE system, the more hydrophilic SC or DOC push the SWCNTs into the more hydrophilic dextran‐rich phase. In contrast, the less hydrophilic SDS moves the SWCNTs into the less hydrophilic PEG‐rich phase. Regarding nonionic surfactants, their use in ATPE separation remains unexplored. We recently observed that, in the ATPE routine, Pluronic F127,^[^ ^15^ ^]^ essentially a PEG derivative, shifts SWCNTs into the PEG‐rich phase, possibly due to similar chemical nature. As a result, (7,5) SWCNTs with notable spectral homogeneity were harvested.

The affinity of anionic SC and DOC to small‐diameter SWCNTs has already been reported many times.^[^ ^10^ ^]^ It was noted that when dispersions prepared in these surfactants are used, the ATPE separation often proceeds according to the diameter cut‐off mechanism. That is, SWCNTs migrate from the lower to the upper phase starting with the ones of the largest diameters as the SDS content increases, leaving smaller species, such as (6,5) SWCNTs, in the bottom fraction.

Conversely, there is a scarcity of data regarding the behavior of nonionic surfactants in various SWCNT differentiation processes. To fill this gap of understanding, we chose Triton X‐100 (TX100) as a model surfactant, which is readily available, widely used, cheap, and well characterized in the literature.^[^ ^32^ ^]^ For sample preparation, a specified volume of TX100 (2.5% or 10% water solution) was introduced to a system consisting of DEX, PEG, DOC, SWCNT dispersion stabilized by bile salt surfactants, and water. Respective amounts of the nonionic surfactant were added to promote SWCNT partitioning, and the missing volume was refilled with water to reach the same sample volume of 4.59 mL each time (Table S3, Supporting Information).

Keeping in mind that DOC has a stronger pushing power to the bottom phase than SC,^[^ ^10^ ^]^ we used a smaller than usual volume of SWCNT dispersion (in DOC) and the DOC solution itself (hoping that the DOC content would not prohibit stepwise extraction of SWCNTs to the top with a nonionic counter‐surfactant). However, the results showed that DOC is such a strong surfactant that a 2.5% solution of TX100 is insufficient to extract most SWCNTs to the top phase across the tested volume range (the extraction system has a constant volume, and the maximum amount of added partitioning modulator is 2.4 mL). Therefore, we also used a 10% TX100 solution (in order not to exceed the assumed constant sample volume). With the increase of TX100 volume, SWCNTs of larger diameters were preferentially and gradually transferred to the top phases (Figure S1, Supporting Information). Unfortunately, no monochiral fraction could be obtained in the bottom phase. Nevertheless, the high content of TX‐100 (10%, 900 µL) enriched the bottom fraction with (6,4) SWCNTs, which appeared promising to us, so we started investigating whether optimization of the parameter space could afford higher purity.

As a side note, we observed that for the 1800 µL addition of 2.5% TX100 in the aforementioned partitioning system, the conditions allowed somewhat effective discrimination between small‐ and large‐diameter SWCNTs. The bottom phases contained mostly (6,4), (6,5), (7,3), (8,3) SWCNTs, while the larger diameter SWCNTs of chiralities such as (7,5), (7,6), (8,4), (9,4), and (10,2) occupied the top phases. We successfully validated that it is possible to obtain them at a large scale and high concentration (Figure S2a,b, Supporting Information) by processing considerable SWCNT suspension volumes. As a result, small‐ and large‐diameter fractions, having vivid colors, with PL emission rich in (7,5) (Figure S2c, Supporting Information) and (6,5) (Figure S2d, Supporting Information) chirality were obtained, which may be useful in applications that do not require monochiral purity.

### 2.3 Partitioning Using SC SWCNT Dispersion and SC/TX100 Surfactant Mixture

**Figure 2:**
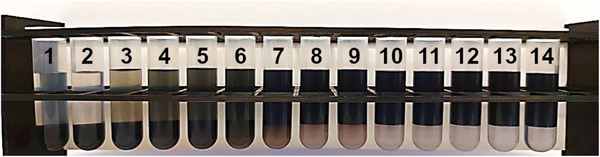
Color changes of ATPE phases with increasing TX‐100 addition from left to right. Conditions for sample preparation are reported in Table S4 (Supporting Information).

Being aware of the weaker SC downward extraction strength compared to DOC, we decided to use it instead so that TX100 could counteract it. We prepared a series of samples with increasing amounts of TX100 (Table S4, Supporting Information). In the absence of a nonionic surfactant, all SWCNTs were in the bottom phase. As the nonionic surfactant volume was increased, a successive shift of SWCNTs to the top phase occurred. This process was observable even with the naked eye. With the increase of the additive, the color of the bottom phase changed from black through purple and pink to faint blue. Simultaneously, the color changed from colorless through green to black for the top phases (**Figure** **2**).

**Figure 3:**
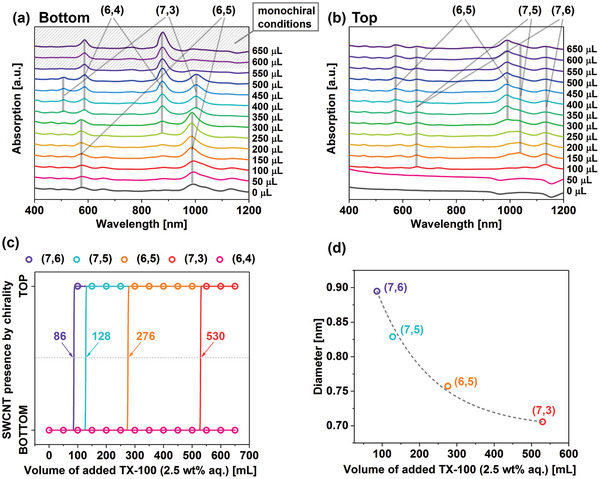
Absorption spectra of SC/TX‐100/SC SWCNT sample series: a) bottom and b) top phases—S_22_ and S_11_ optical transitions of major semiconducting species were indicated. c) Estimation of SWCNT phase transition points based on the absorption data enclosed in panels (a) and (b). The lowest TX100 volume at which a specific SWCNT chirality appeared in the top phase or disappeared from the bottom phase was noted, and the SWCNTs were assigned to the top phase. Then, the obtained data were fitted with a step function, and TX100 volumes at mid‐heights of the transition lines were extracted. d) Recorded mid‐height TX100 volumes were correlated with the diameter of SWCNTs transiting from the bottom to the top.

The dynamic color change of the samples suggested that the SWCNTs migrated in some particular order, which should be elucidated. These observations were analyzed in detail using absorption spectroscopy (**Figure** **3**). In the bottom phases, initially (0–50 µL of 2.5% TX‐100), all nanotube types were present (Figure 3a). Next, a further increase in TX100 (100–300 µL) gradually removed large diameter SWCNTs ((7,6), (10,2), (7,5)) from the bottom phases, paving the way for the migration of the small diameter SWCNTs such as (6,5), (6,4), or (7,3). Once 350 µL TX100 volume was reached, the peak in the ≈1000 nm region appeared to be shifted to the longer wavelengths. A corresponding feature at ≈510 nm emerged, suggesting a considerable presence of (7,3) SWCNTs rather than (6,5), which diffused to the top phase. Subsequently, even higher TX100 concentration made (6,4) SWCNTs abundant in the bottom phases. Beyond 500 µL, the bottom phases seemed to be composed entirely of (6,4) SWCNTs. Concomitantly, with the increase in TX100, the top phases become richer in (7,6), (7,5), and (6,5) SWCNTs (Figure 3b). Therefore, the addition of TX100 discriminated the SWCNTs by diameter, gradually shifting the SWCNTs with the largest diameters into the top phase, leaving the smallest species in the bottom. Interestingly, at high TX100 concentrations, the top phases’ spectra resembled the parent SWCNT dispersion, but they did not contain (6,4) SWCNTs. It appeared that the TX100 molecules, which shifted SWCNTs from the bottom to the top, somehow could not effectively bind to (6,4) SWCNTs. Furthermore, TX100 concentrations at which the most SWCNTs migrated to the top phase were established for four SWCNT chiralities (Figure 3c). Comparison of these established TX100 concentrations revealed a striking exponential correlation with the diameter of the separated SWCNT types (Figure 3d). This relation may be used to estimate the conditions of separation that are necessary to enable isolation of targeted SWCNT types.

**Figure 4:**
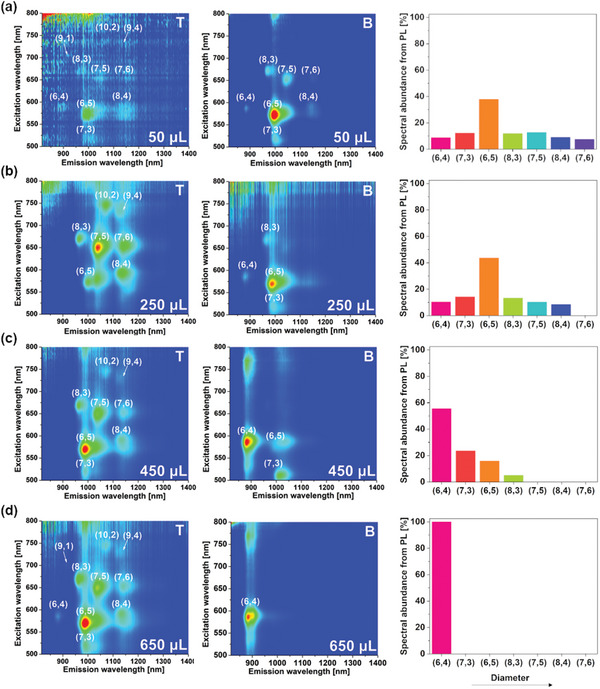
PL excitation‐emission maps of top and bottom samples of increasing TX100 volume: a) 50, b) 250, c) 450, and d) 650 µL of 2% TX100 aqueous solution. The abundance of particular SWCNT species was estimated from PL excitation‐emission maps by comparison of peak maxima intensities.

PL excitation‐emission mapping was engaged to probe the optical purity of the separated samples and confirm successful separation (**Figure** **4**). The obtained results stayed in accordance with previously discussed absorption data. As the TX100 concentration increased, large‐diameter SWCNTs were shifted to the top phase, which eventually gave rise to the emergence of a (6,4) monochiral fraction in the bottom (650 µL addition of TX100 solution). Registered PL originated exclusively from (6,4) SWCNTs, demonstrating remarkable selectivity of the reported technique.

**Figure 5:**
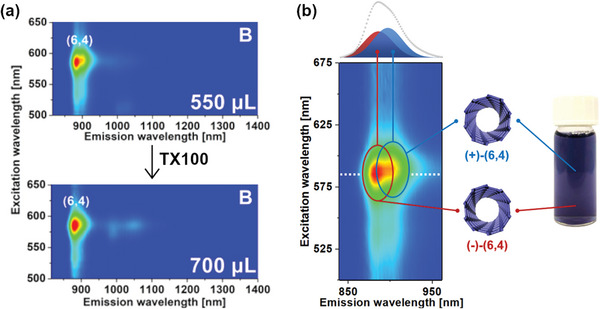
a) PL excitation‐emission maps of bottom samples of different TX100 volumes. b) Magnification of the 550 µL TX100 sample showing enantiomeric resolution of (6,4) SWCNTs (the top part demonstrates deconvolution of the PL spectrum into two peaks corresponding to (6,4) SWCNT of different handedness).

At this point, the goal was to fine‐tune the conditions, which enabled the isolation of highly pure (6,4) SWCNTs to determine the optimum purification conditions more accurately (Figure S3, Supporting Information). Only (6,4) SWCNTs were detectable in the bottom phase already at 550 µL of TX100, and even when the TX100 volume was increased to 700 µL, (6,4) SWCNTs remained in the bottom, confirming that the shift of these species to the top phase was still not favorable under these conditions. Interestingly, a detailed investigation of the recorded spectra revealed very clearly the presence of two (6,4) SWCNT enantiomers when 550 µL of TX100 was added (**Figure** **5**). At the higher TX100 content (700 µL) (+)‐(6,4) SWCNTs migrated to the top phase, leaving the (−)‐(6,4) SWCNT‐rich fraction in the bottom.

A closer examination of the PL excitation‐emission data supplied evidence supporting the hypothesis of the presence of two optical isomers.^[^ ^33^ ^]^ Besides the shift of the E_11_ maximum of (6,4) SWCNTs, the E_11_* peak signaling defects,^[^ ^34^ ^]^ likely introduced during sonication^[^ ^35^ ^]^ due to the high reactivity of small‐diameter SWCNTs, was of different intensity in these two samples (Figure S4, Supporting Information). It was previously observed that (6,5) SWCNTs dispersed with DNA exhibit much different reactivity depending on the handedness when they are oxidized with NaClO.^[^ ^36^ ^]^ This effect was ascribed to the dissimilar coverage of these two forms of SWCNTs with DNA. In our case, the chiral sodium cholate can also coat the left‐ and right‐hand isomers of (6,4) SWCNTs differently, which justifies why the content of defects in these two species was not the same.

### 2.4 Partitioning Using Different SWCNT Dispersions

Importantly, the developed protocol allowed the extraction of chirality (6,4) not only from the SG65i material, which is rich in small‐diameter SWCNTs, but also from other commercially available SWCNTs such as HiPco (average diameter distribution of 0.8–1.2 nm^[^ ^37^ ^]^) and SG76 (average diameter: 0.9 nm^[^ ^38^ ^]^). Particular attention should be paid to HiPco material, where (6,4) chirality appears to be outside the expected species range (Figure S5, Supporting Information), yet it was isolated using the established sorting conditions. Thus, the proposed method is universally selective regardless of the type of raw SWCNTs processed.

Furthermore, to align with the zero waste principles, we wanted to validate whether (6,4) species can be harvested equally efficiently from spent SWCNTs. The literature shows that every time SWCNT dispersions are prepared by sonication or shear‐mixing, the homogenized mixture is centrifuged, and only the individualized SWCNTs in the supernatant are collected for experiments. In contrast, the sediment located at the bottom of the vials composed of bundled SWCNTs is simply discarded. Given the price of widely researched (6,5)‐enriched SWCNTs (currently >1 200 USD per gram excluding VAT), sacrificing 10–20% of SWCNTs appears unpractical. Over the past years, we accumulated these solid residues, and for the sake of this study, we decided to test if such a source of SWCNTs could be used for the separation. The bundled SWCNTs, without introducing any additional SC surfactant, were sonicated and centrifuged. The obtained new supernatant was injected into the ATPE system modulated by SC and TX‐100 as in previous experiments. As a result, (6,4) SWCNTs of excellent purity emerged in the bottom phase (Figure S6, Supporting Information). Moreover, the color of the bottom phase was highly intense, proving that the concentration of the harvested species is substantial. Hence, the commonly neglected SWCNTs are equally valuable as fresh SWCNT material.

Another peculiar finding was that after several hours or days, each top fraction experienced precipitation. Fine SWCNTs were materialized out of the otherwise clear suspension, which could be redispersed by manual shaking or short bath sonication. For the lower fractions, the situation was not evident. These observations shed some light on the mechanism of the separation. As described above, TX100 is responsible for shifting SWCNTs from the bottom to the top phase, similar to Pluronic, which is another nonionic surfactant.^[^ ^15^ ^]^ Therefore, the destabilization of SWCNTs in the top phase may result from the exchange of bile salt surfactant for TX100 on the SWCNT surface. Since nonionic surfactants do not individualize SWCNTs equally well as anionic surfactants,^[^ ^39^ ^]^ solute precipitation can be expected. To confirm this reasoning, a spectrum of the SWCNT dispersion prepared using TX100 is given in Figure S7 (Supporting Information). The results showed poor individualization of SWCNTs (compared to SWCNT suspensions made using DOC or SC), which validated this hypothesis.

In light of the above‐described dynamic exchange of surfactant molecules on the SWCNT surface, it was necessary to find out if it is possible to program the ATPE separation outcome already at the individualization step. Based on the successful extraction parameters of pure (6,4)‐chirality (650 µL of 2.5% TX100 addition; 4.59 mL sample volume), the total amount of SC and TX100 in the system was calculated, and a binary surfactant dispersion via a direct combination and sonication of these components in water was prepared. 1 mL of such dispersion contained the same amount of SWCNTs, SC, and TX100 as the regular sample produced by the ATPE, which previously provided the highest degree of monochiralicity. The prepared binary surfactants dispersion (1 mL) was mixed with 1 350 µL DEX solution (20% in water), 540 µL PEG solution (50% in water), and 1 700 µL of DI water. After centrifugation and separation, the obtained two phases were characterized by PL excitation‐emission mapping (Figure S8, Supporting Information). To our delight, the composition of the two phases matched those obtained by typical ATPE processing. (6,4) SWCNTs were exclusively isolated in the bottom phase, whereas the remainder occupied the top phase. The prepared binary surfactant dispersion confirmed that the effective substitution of SC for TX100 on the SWCNT surface determines whether the ATPE purification is effective. It should be noted that the new approach (Figure S9, Supporting Information) based on two surfactants is scalable and may be applicable for processing hundreds of milliliters of SWCNT suspensions at a time, even by nonspecialists. Hence, it should make chirality‐sorted SWCNTs more abundant, facilitating progress in this field.

### 2.5 Partitioning Using Different Nonionic Surfactants as Modulators

One may wonder if other nonionic surfactants would behave equally well as Triton X100. The results showed that appropriate concentrations of several different nonionic surfactants, such as Brij C10, Brij O10, Brij O‐20, Tween‐80, and Genapol X‐080), also produced fractions highly enriched with (6,4) SWCNTs or even monochiral (Figure S10, Supporting Information). The results for linear Brij‐35 and Tween‐20, a sorbitan derivative, are presented in the main manuscript. For processing conditions and results in a broader range using the mentioned surfactants, please refer to Tables S5 and S6, and Figures S12 and S13 (Supporting Information). Both compounds have substantially different structures compared to TX100. Still, after parameter optimization, each surfactant allowed the extraction of pure (6,4)‐SWCNTs in the bottom phase, using the SC/SWCNT dispersion and SC as a counter‐surfactant (Figure S11, Supporting Information). Regardless of the surfactants used, the end results of the separation were similar, which begs the question of what is the underlying ATPE mechanism when nonionic surfactants are involved.

### 2.6 Elucidation of the Mechanism of the Separation

The key properties of employed surfactants (Table S7, Supporting Information) were compared to solve this conundrum. All nonionic surfactants had a similar HLB value, and their concentration under conditions leading to effective (6,4)‐SWCNT chirality extraction exceeded their CMC. Furthermore, average micellar weight was quite divergent, so micelle characteristics should not be the main factor to consider unless the key was to reach a certain threshold. Also, regarding the chemical structure, the end groups were much different. Similarly, the ethoxy unit content with respect to the total weight of the surfactant varied widely between these compounds. Finally, the approximate concentration of nonionic surfactants leading to satisfactory separation of (6,4)‐chirality in the bottom phase differed substantially as well. As deducing the extraction mechanism using characteristics of the employed nonionic surfactants exclusively was unsuccessful, the experimental study was complemented with modeling to unravel the interactions between surfactants and SWCNTs.

### 2.7 Molecular Dynamics Simulations of Surfactant Adsorption

**Figure 6:**
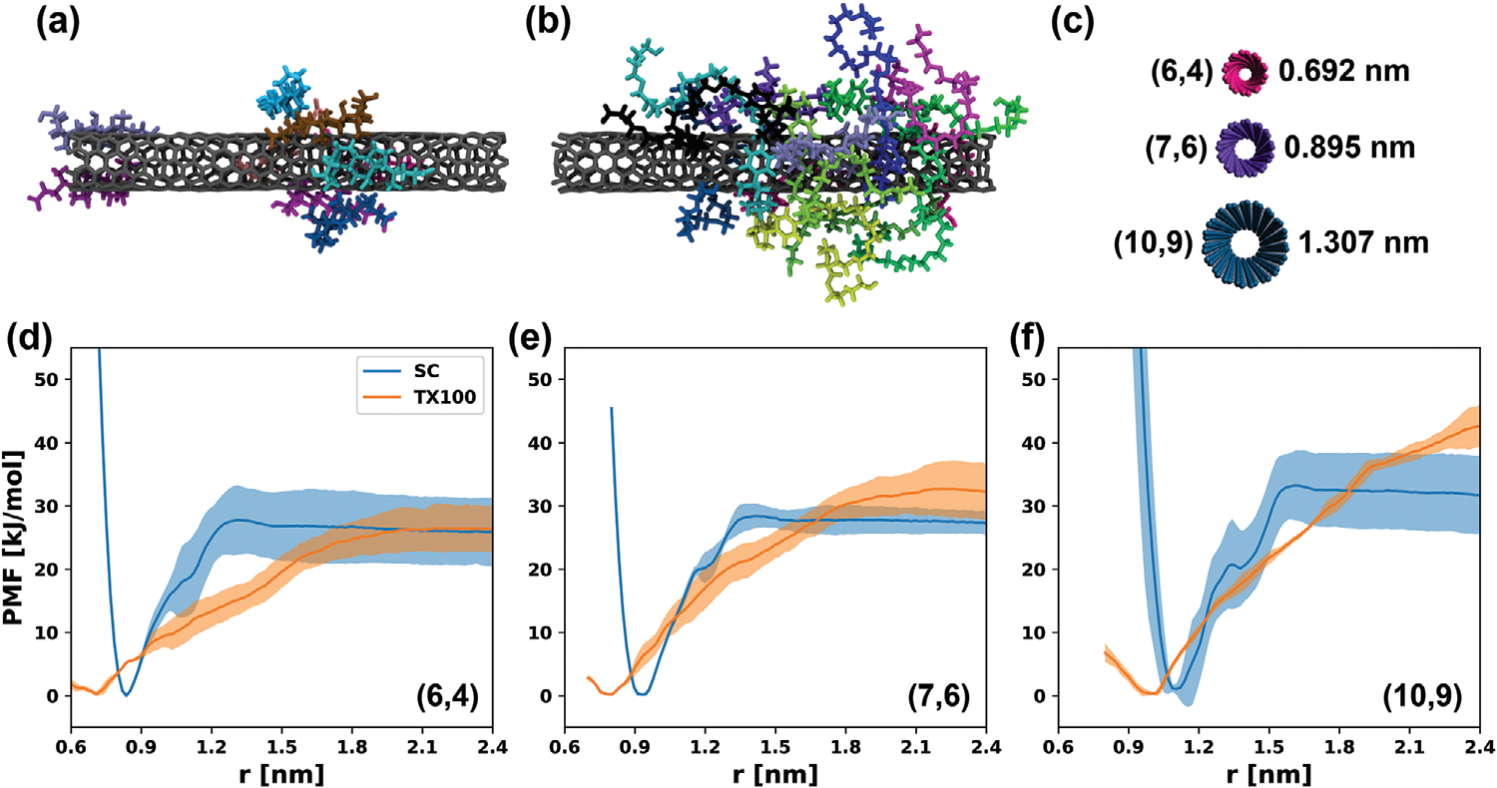
Multiple surfactant molecules equilibrating for 10 ns on the surface of the (6,4) SWCNT: a) SC, and b) TX100. c) Difference in size of investigated SWCNTs. PMF profiles for adsorption of single surfactant molecules on the d) (6,4) SWCNT, e) (7,6) SWCNT, and f) (10,9) SWCNT. Data obtained via umbrella sampling and the weighted‐histogram analysis method (WHAM). The minimum of each energy profile has been defined as zero for visualization purposes. Each profile is the average of 4 individual US runs and the uncertainty corresponds to one standard deviation.

Molecular dynamics simulations were used to assemble surfactant corona phases on SWCNT surfaces at various bulk surfactant concentrations. Examples of such coronas can be seen in **Figure** **6**a,b for SC and TX100 surfactants, respectively. Our simulation study encompassed three different SWCNT types (Figure 6c) to determine the effect of larger diameters on several adsorption characteristics. We first examined the adsorption energy of a single surfactant molecule with a bare SWCNT via umbrella sampling (US). The resulting potentials of mean force (PMFs) (Figure 6d–f) indicate that the adsorption energy of SC is independent of SWCNT diameter (≈30 kJ mol^−1^ for all three SWCNTs), while the TX100 molecule shows preferential binding (higher adsorption energies) to larger diameter SWCNTs (≈40 kJ mol^−1^ with (10,9) SWCNTs). This can be attributed to the fact that the SC molecule is more rigid and compact on the SWCNT surface and its adsorbed conformations are not considerably affected by changes in curvature. Contrastingly, the flexible tail of the TX100 imparts much more conformational diversity and can wrap around the SWCNT differently depending on the curvature present. Similar results (higher adsorption energies to large‐diameter SWCNTS) were observed when examining Brij‐35 as an alternate nonionic surfactant with a flexible tail (Figure S14, Supporting Information), the use of which also produced monochiral fractions of (6,4) SWCNTs.

**Figure 7:**
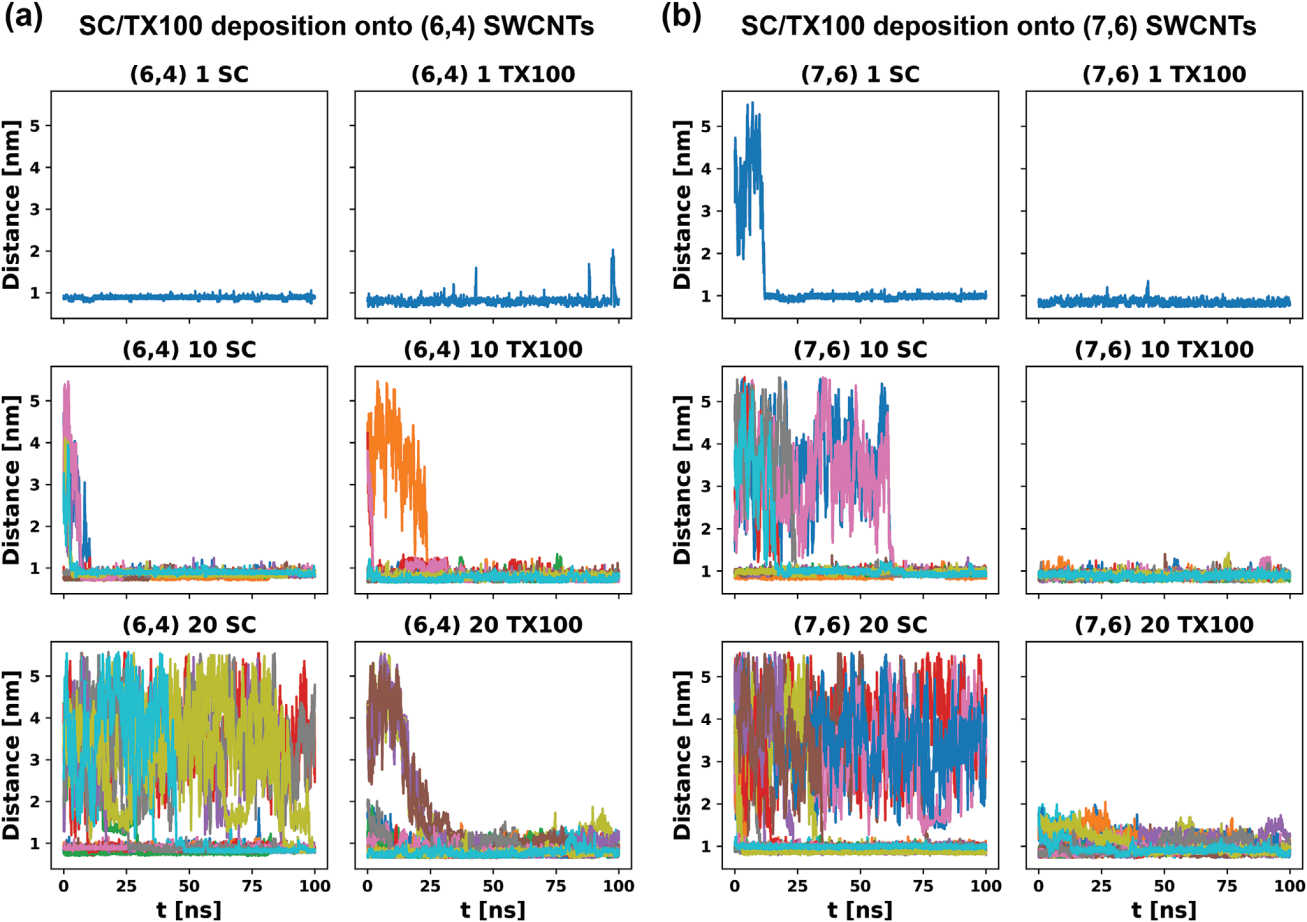
COM distance of individual surfactant molecules (*N* = 1, 10, and 20 molecules of SC or TX100) from SWCNT axis as a function of time for a) (6,4) SWCNTs, and b) (7,6) SWCNTs. Distances less than 2 indicate adsorption of that particular molecule.

Following the energetic analysis of the single surfactant molecule, we assembled different surfactant corona systems consisting of 10, 20, and 40 surfactant molecules. This assembly process was not directed and was allowed to occur naturally due to attractive interactions between the surfactant and the SWCNT. Analysis of the length of time needed to fully assemble the various corona phases (**Figure** **7**; and Figure S16, Supporting Information) demonstrate that in general, the TX100 corona forms more readily than that of the SC (most likely due to anionic repulsion). This is most detectable in the case of *N* = 20 surfactant molecules where the TX100 undergoes full adsorption in less than 50 ns, while the SC is still not fully adsorbed following 100 ns of simulation time. See the Supporting Information for a more detailed discussion of how these individual distances were calculated for each surfactant.

**Figure 8:**
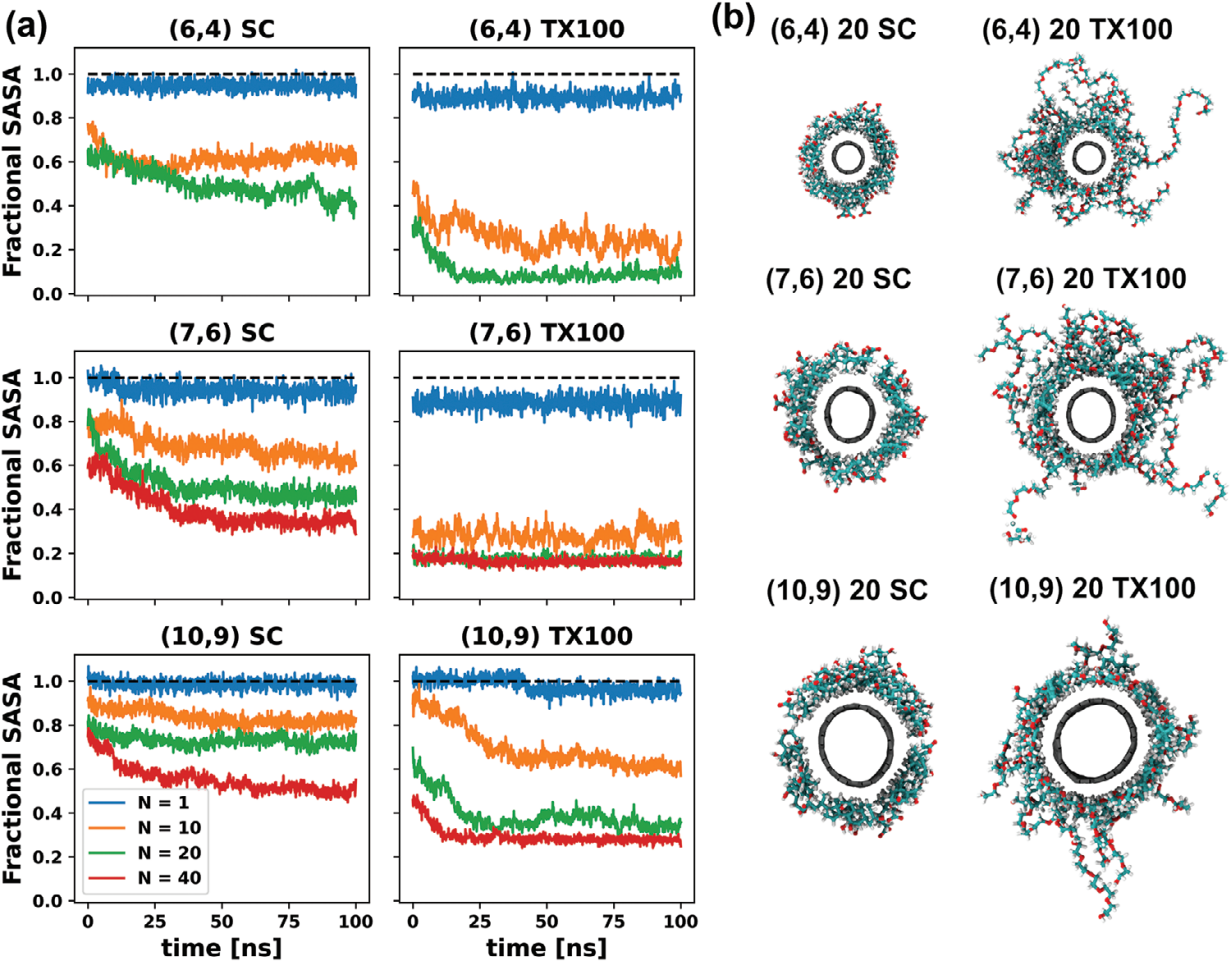
a) Fractional SASA values for the (6,4), (7,6), and (10,9) SWCNTs suspended using *N* = 1, 10, 20, and 40 molecules of (left) SC, and (right) TX100. The dashed line denotes the fractional value of 1.0, i.e., a fully bare SWCNT. b) Snapshot of the surfactant/SWCNT complex for specified systems.

Equilibrium of this corona formation was monitored via the fractional solvent accessible surface area (SASA) of the SWCNT (a measure of what fraction of the SWCNT surface is not covered by surfactant molecules). **Figure** **8**a reveals that, in general, a higher fraction of the SWCNT is covered with a TX100 corona compared to an SC corona made up of the same number of molecules. While this is not necessarily surprising due to the TX100 molecule being larger than SC, it does reinforce the observation that the SWCNT surface (especially at larger diameters) more readily interacts and adsorbs nonionic surfactant corona phases. The same SASA data are presented in absolute form in Figure S17 (Supporting Information). Representative snapshots of all 20 molecule surfactant coronas are displayed in Figure 8b (snapshots of all corona systems assembled can be seen in Figure S18, Supporting Information).

Once these corona phases were assembled, further analysis was carried out to determine the level of cooperativity in the adsorption of multiple surfactant molecules on the SWCNT surface. This was carried out via another round of US simulations, but this time the PMF was obtained by removing a single surfactant molecule from the pre‐assembled corona (visualized in Figure S19, Supporting Information). Results indicate that on the small diameter (6,4) SWCNTs (Figure S19, Supporting Information), the adsorption is cooperative for both SC and TX100 surfactants (adsorption energies ≈30 kJ mol^−1^ to remove a single SC molecule from a bare SWCNT increasing to ≈40 kJ mol^−1^ to remove a single molecule from a corona of *N* = 20). Consequently, replacing SC molecules on the surface of (6,4) SWCNTs, which keep them in the bottom phase, with TX100 to shift them to the top phase, is energetically challenging. On the larger (7,6) SWCNT (Figure S20, Supporting Information), the cooperativity is less apparent for SC (the adsorption energy is within the uncertainty margin for all *N* = 1, 20, and 40), but still present for TX100 (displaying an increase in adsorption energy at *N* = 40). This increase in cooperativity on the larger‐diameter SWCNTs is yet another measure of the preference for the corona formation of nonionic surfactants over SC.

Finally, in addition to the previously described energetic observations, we also quantified various structural metrics of the different corona phases. Details of this structural analysis can be found (Figures S21–S29, Supporting Information). We find that the conformations assumed by the SC molecules within the corona are much more limited than those of the TX100. This is again most likely due to the high flexibility of the long‐chain tail and the lack of repulsive anionic interactions between the adsorbing surfactant molecules. Furthermore, our findings indicate that the TX100 corona phases much more readily displace water from the SWCNT surface, as shown in Figure S22 (Supporting Information) (this is in line with our SASA results). As the top phase is more hydrophobic, the observed water removal explains why nonionic surfactants favor the shift of SWCNTs to the top phase. Finally, the analysis of the orientation of surfactant molecules with respect to the SWCNT axis (Figure S29, Supporting Information) reveals that SC molecules arrange differently depending on whether the dihedral angle between the molecular vector and the nanotube axis is positive or negative. This effect is particularly notable in the case of (6,4) SWCNT, for which the part of the histogram covering the positive angles has, on average, higher intensity and different lineshape than that of the negative ones. The highlighted discrepancy confirms that the chiral SC molecule has a different affinity to left‐ and right‐handed optical isomers of (6,4) SWCNTs, leading to their dissimilar representation in solution (Figure 5; and Figure S4, Supporting Information). Therefore, the combination of SC with nonionic surfactants enables high‐resolution partitioning of SWCNTs, the mechanism of which is illustrated in Figure S30 (Supporting Information).

## 3 Conclusion

In summary, we demonstrated highly effective separation of (6,4)‐SWCNT chirality in one step by the ATPE method. The developed method presented exceptional isolation resolution, enabling harvesting of monochiral fraction of the mentioned specie from many types of polydisperse mixtures of SWCNTs, including commonly generated SWCNT waste. Because of the simplicity, robustness, and scalability of the reported approach, the results pave the way for the broad‐scale utilization of small‐diameter SWCNTs also by nonspecialists. The isolated SWCNTs have considerable application potential due to their notable reactivity and valuable optoelectronic properties, which should catalyze progress in the field.

Through thorough experimentation, we determined that successful differentiation can be achieved using several nonionic surfactants (Triton X‐100, Brij‐35, Tween‐20, etc.) instead of commonly employed anionic surfactants such as sodium deoxycholate and sodium dodecyl sulfate. We observed that nonionic surfactants have a higher affinity to large‐diameter, so they can be used for extraction of such SWCNT types, leading to the concentration of (6,4) SWCNTs in the bottom phase. Molecular dynamics simulations revealed that, while the adsorption energy of bile salt surfactants is independent of SWCNT diameter, nonionic surfactants exhibit preferential binding (higher adsorption energies) to larger diameter SWCNTs. Moreover, analysis of surfactant corona phases highlighted that nonionic surfactants exhibit increased levels of cooperative adsorption and displace water from the SWCNT surface more readily, thereby underlying the importance of supramolecular organization and unraveling the mechanism of the ATPE separation approach devised in this study. Due to the universal nature of the discovered relations between SWCNTs and nonionic surfactants, the gained insight can facilitate the development of more effective processing strategies for a wide scope of nanomaterials, especially since the full potential of nonionic surfactants in such processes has not been unleashed yet. It is likely that previously unexplored combinations of nonionic and ionic surfactants, when implemented in ATPE, chromatography, or density gradient ultracentrifugation, may give rise to superior sorting results.

## 4 Experimental Section

### Materials and Reagents

Dextran (DEX, *M*_R_ ≈70 kDa, PanRecAppliChem, Germany), poly(ethylene glycol) (PEG, *M*_n_ ≈6000 Da, Alfa Aesar, Germany), sodium cholate (SC, PanRecAppliChem, Germany), sodium deoxycholate (DOC, Sigma‐Aldrich, USA), Triton X100 (TX100, Thermo Fisher Scientific, USA), Brij 35 (Sigma‐Aldrich, USA), Tween‐20 (VWR, USA), Brij O20 (Sigma‐Aldrich, USA), Brij O10 (Sigma‐Aldrich, USA), Genapol X080 (Sigma‐Aldrich, USA), Brij C10 (Sigma‐Aldrich, USA), Tween‐80 (VWR, USA), and sodium hydroxide (Chempur, Poland) were all of pure p.a. class, so they were employed without any further purification. For this work, Signis SG65i SWCNTs (Sigma‐Aldrich, LOT: MKCK1004) enriched in (6,5) chirality to simplify the interpretation of SWCNT migration between the phases due to the reduced number of species. Wherever specified, HiPco (Nanointegris, Canada; LOT: HP30‐006, purified) and Signis SG76i (Sigma‐Aldrich, LOT: MKBZ1157V) SWCNTs as references were also used to investigate the ATPE mechanics. For all sorting experiments and characterization, double‐distilled water obtained from the Elix Millipore system was used.

### Preparation of SWCNTs Dispersions

Regardless of the starting material (unsorted or rich in (6,5)/(7,6) SWCNTs), the same protocol of sonication was used. First, freshly prepared aqueous surfactant solution (2% w/w; 40 mL) and SWCNTs powder (40 mg) were introduced into a 50 mL vial. Then, the mixture kept in an ice bath was sonicated (Hielscher UP200St, 200 min, 30 W). Subsequently, the suspension was centrifuged (Eppendorf Centrifuge 5804 R) at 18 °C at the Relative Centrifugal Force (RCF) of 15314 × g for 1.5 h to remove the nonindividualized SWCNTs. The upper 80% of supernatants were collected and used in the experiments described below.

To prove that the always disposed of sediment from the SWCNT sonication/centrifugation process can also be a valuable source of SWCNTs, it was used as a source of SWCNTs for purification as well. Solid deposits from the sonication of SWCNTs with SC accumulated over the past 3 years were sonicated and subjected to centrifugation using a similar procedure. No extra surfactant was added to facilitate homogenization. Analogously, 80% of generated supernatant was used for this study, while the remaining part was preserved for future experiments.

The same sonication and centrifugation conditions for SWCNT dispersion facilitated by a binary mixture of surfactants was used. In this case, the suspension was prepared from 8.4 mg SWCNT powder introduced into a combination of sodium cholate solution (10%; 5.15 mL), Triton X100 solution (10%; 15.15 mL), and water (17.10 mL). All prepared SWCNT dispersions were black, and no aggregates were observed, confirming the high concentration of the SWCNT dispersions and their successful individualization.

### Density Measurement

Density was determined using a pycnometer. All stock solutions were examined at the same conditions, at room temperature. First, the calibrated pycnometer was incubated at room temperature with the discussed stock solutions. Then, ≈2 mL of the solution was pipetted into the pycnometer, and the outlet was closed with a stopper. The extruded liquid droplets were carefully rubbed off, and the entire set was placed on an analytical balance to note the weight. After the measurement, the vessel was washed with deionized water and acetone. Another reading was done after the clean glass was dry and its temperature was stabilized.

### ATPE Protocol

Stock solutions (Table S1, Supporting Information) of PEG, DEX, surfactants, DI water, and SWCNT dispersion (according to the mentioned order) were added to a round‐bottom centrifuge tube (6 mL), and the mixture was gently homogenized by a Vortex mixer (about 10 s per sample). Then, the obtained suspensions were centrifuged (Eppendorf Centrifuge 5804 R) for 3 minutes at 18 °C at the RCF of 2025 × g, which promoted phase separation. The mixtures that split into two phases were immediately harvested by pipetting. The same protocol was used for SWCNT dispersion made using two surfactants, with the difference that no surfactants stock solutions were used in such a case during separation. The general parameters used for ATPE processing are summarized in Table S2 (Supporting Information).

### Optical Characterization

UV–VIS–NIR spectra were measured with the PerkinElmer Lambda 1050 spectrophotometer. Artifacts located near 860 nm at UV–VIS–NIR spectra are related to detector change. Excitation‐emission photoluminescence (PL) maps were recorded across the specified wavelengths ranges: excitation (500–800 nm) and emission (800–1400 nm) using Horiba Jobin Yvon spectrofluorometer (Fluorolog‐3 with FluorEssence).

### Simulation Setup

All simulations were carried out using GROMACS (ver. 2021.2)^[^ ^40^ ^]^ applying the standard TIP3P model for water^[^ ^41^ ^]^ and the CHARMM36 forcefield parameters^[^ ^42^ ^]^ for the cholate ion. The forcefield parameters for TX100 were taken from Yordanova et al.^[^ ^43^ ^]^ Coordinate files for the two surfactants were obtained from the CHARMM‐GUI small molecule library,^[^ ^44^ ^]^ and those for the SWCNTs were generated using VMD.^[^ ^45^ ^]^ The SWCNTs were modeled using the standard CHARMM36 parameters for *sp* ^2^‐carbons and were constructed as infinitely long objects that crossed the periodic boundary of the system in the z‐direction. Each nanotube was placed in a box 10 nm wide in both the x‐ and y‐dimensions. The z‐dimension of the system was determined by the corresponding unit cell length for each SWCNT—5.57 nm for (6,4) (corresponding to 3 unit cells), 4.8 nm for (7,6) (corresponding to one unit cell), and 7.11 nm for (10,9) corresponding to one unit cell).

### Corona Assembly

In each case, the appropriate number of the surfactant of interest was randomly inserted into the box, and then the SWCNT/surfactant system was solvated. Na^+^ ions were also included in the SC systems to maintain charge neutrality. Following energy minimization, the thermostat of Bussi et al.^[^ ^46^ ^]^ was used to thermalize the system for 250 ps in the NVT ensemble. The system was then allowed to relax for 250 ps in the NPT ensemble applying the Parrinello–Rahman barostat.^[^ ^47^ ^]^ This was followed by a 10 ns equilibration run, and the radial distance between the SWCNT axis and the surfactant center‐of‐mass (COM) was observed. In the case of TX100, adsorption to the SWCNTs happened quickly (< 7.5 ns). Single cholate ions did not adsorb during the 10 ns simulation time, so to speed equilibration, a harmonic restraint was added to pull the cholate to the SWCNT surface over the subsequent 2 ns. The harmonic restraint was then removed, and the adsorbed cholate was allowed to equilibrate for an additional 10 ns. For the systems containing multiple surfactant molecules, an additional 100 ns of simulation time was undertaken to allow for the assembly of the corona structure. Equilibration of these structures was monitored via the solvent‐accessible surface area of the SWCNT. Three equally spaced snapshots from each system were taken from the final 5 ns of the equilibration trajectory and used as starting configurations for umbrella sampling.

### Umbrella Sampling

Umbrella sampling (US) was performed with a window spacing of 0.4 Å. The number of windows was dependent on the surfactant species being investigated. The smaller, more compact SC molecules were more readily removed from the surface (60 windows), while the long, flexible tails of TX100 and Brij‐35 required a further separation distance to achieve complete surface desorption (80–100 windows). The US was carried out by restraining the radial distance between the SWCNT axis and the surfactant COM using a harmonic potential having a spring constant of *k* = 10^4^ kJ mol^−1^ nm^−2^. This resulted in sufficient overlap between the histograms of the sampled configurations. The weighted‐histogram analysis method (WHAM),^[^ ^48^ ^]^ as implemented in the *g_wham* module^[^ ^49^ ^]^ within GROMACS, was then engaged to construct the energy profiles. For comparison purposes, the minimum value of each profile was translated to zero^[^ ^50^ ^]^.

## Conflict of Interest

The authors declare no conflict of interest.

## Supporting information

Supporting Information

## Data Availability

The data that support the findings of this study are available from the corresponding author upon reasonable request.
