# Supplementary material for: Highly‐Selective Harvesting of (6,4) SWCNTs Using the Aqueous Two‐Phase Extraction Method and Nonionic Surfactants
Source: Adv Sci (Weinh). 2023 Mar 1;10(14):2207218. doi: 10.1002/advs.202207218 (PMC10190623; doi:10.1002/advs.202207218)
Supplement: Supplementary file 1 — Supporting Information [file ADVS-10-2207218-s001.pdf]

Supporting Information

Highly-Selective Harvesting of (6,4) SWCNTs Using the Aqueous Two-Phase Extraction Method and Non-Ionic Surfactants

Blazej Podlesny, Kevin R. Hinkle, Keita Hayashi, Yoshiaki Niidome, Tomohiro Shiraki, and Dawid Janas,**

Table of Contents

1. Parameters of ATPE Systems Used in the Study 3
2. Partitioning Using DOC SWCNT Dispersion and DOC/TX100 Surfactant Mixture 4
3. Partitioning Using SC SWCNT Dispersion and SC/TX100 Surfactant Mixture 6
4. Separation of a Spectrum of Different SWCNT Dispersions 8
5. Elucidation of the Mechanism of the Separation 13
   5.1. Calculations 13
   5.2. Simulations 15
      5.2.1. Calculation of Surfactant/SWCNT Distances 15
      5.2.2. Corona Structure 21
   5.3. Mechanism of the Partitioning Process 28
6. References 29

1. Parameters of ATPE Systems Used in the Study

Table S1. Concentration of stock solutions.

Compound	Concentration of stock solution (w/w; water) [%]
DEX (70 kDa)	20
PEG (6 kDa)	50
SC	10
DOC	5
TX-100	2.5 or 10
Brij35	5
Tween20	5
NaOH	28.5

Table S2. General composition of samples classified accordingly to the series of experiments.

Compound	Volume for separated samples made from fresh DOC/SWCNT dispersion [µL]	Volume for separated samples made from fresh SC/SWCNT dispersion [µL]	Volume for samples made from waste sediments generated by previous sonication of SC with SWCNTs [µL]	Volume for separated samples made from binary surfactant dispersion of SWCNTs [µL]
DEX	1 350	1 350	1 350	1 350
PEG	540	540	540	540
SC	-	360	315	-
DOC	150	-	-	-
SWCNT dispersion	150	225	450	1 000
Surfactant (TX100/Brij35/Tween20)	225 – 2 175	50 – 650	650	1 700
Water	0 – 2 400	1 465 – 2 065	1 285	-
Total volume	4 590			

2. Partitioning Using DOC SWCNT Dispersion and DOC/TX100 Surfactant Mixture

Table S3. DOC/TX-100/DOC-SWCNT samples series: composition and description. Each sample contained 1 350 µL of DEX (70 kDa, 20%), 540 µL of PEG (6 kDa, 50%), 150 µL of DOC (5%), and 150 µL of SWCNT dispersion (2% DOC; 1 mg/mL of SWCNTs). Total volume: 4 590 µL. Conditions from the shaded row served during scale-up experiments presented in **Figure S2**.

**TX-100 [µL]**	**DI water [µL]**	**Bottom phase color**	**Top phase color**
0	2 400	Black	Colorless
225 (2.5%)	2 175	Black	Colorless
450 (2.5%)	1 950	Black	Colorless
600 (2.5%)	1 800	Black	Colorless
900 (2.5%)	1 500	Black	Colorless
1 200 (2,5%)	1 200	Black	Colorless
1 500 (2.5%)	900	Black/Purple	Green
1 800 (2.5%)	600	Black/Purple	Green
2 400 (2.5%)	0	Purple	Green
900 (10%)	1 500	Pink	Green/Black

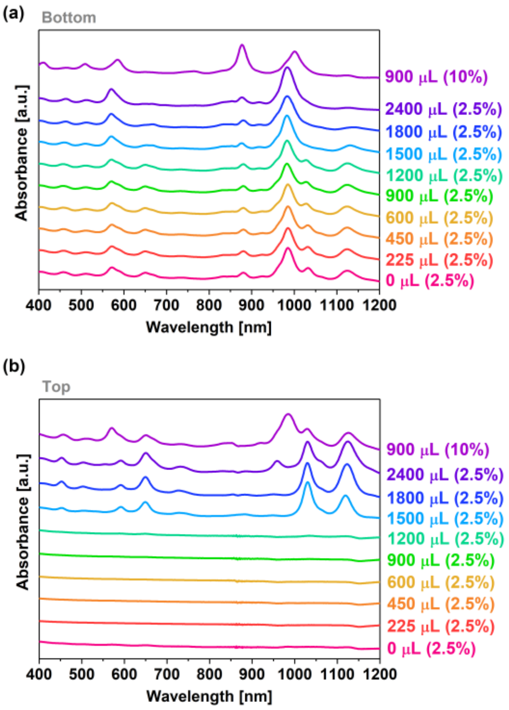

Figure S1. Absorption spectra of (a) bottom and (b) top phases after separation of SWCNTs dispersed in water with DOC at varying TX-100 content.

As stated in the manuscript, DOC pushes SWCNTs to the bottom phase much more strongly. According to our experience, it is perhaps the most potent surfactant modulating partitioning of SWCNTs in the ATPE approach. Thus, upward extraction of DOC-dispersed SWCNTs to the top phase with non-ionic surfactants is very challenging. Consequently, we could not afford monochiral (6,4) SWCNTs in the bottom phase across the explored partitioning conditions. We did not continue increasing TX100 concentration, which was already high, as an excessive amount of TX100 in the presence of DEX may lead to the formation of a DEX/TX100 biphasic system[1]. To avoid these problems, we decided to use SC which was found easier to counteract, i.e. less non-ionic surfactant is required to promote migration of SWCNTs to the top phase.

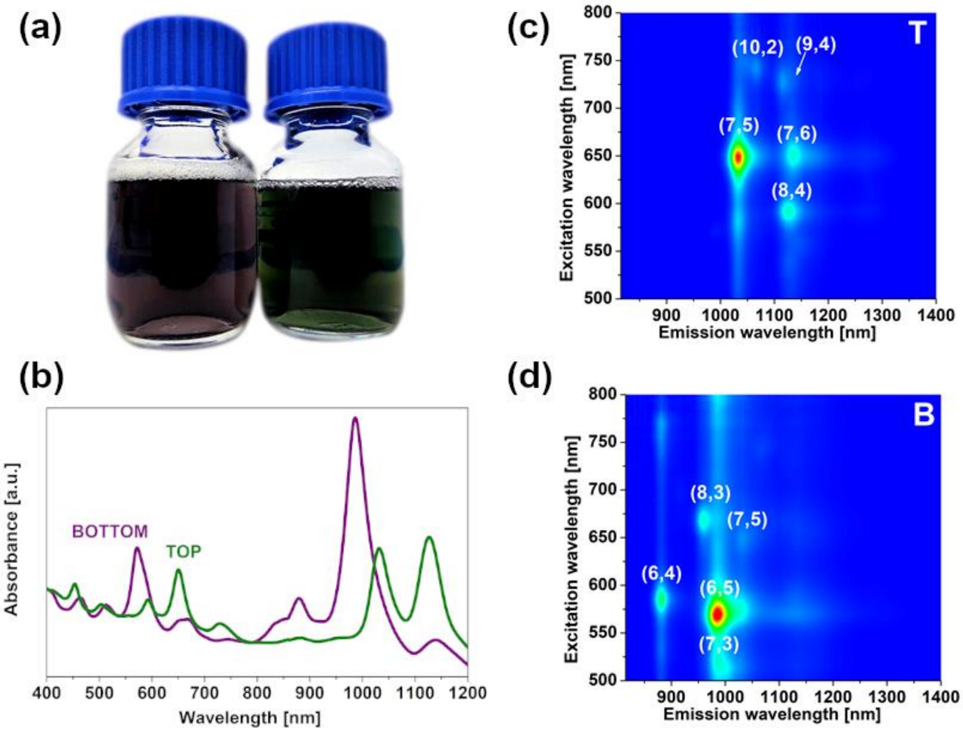

Figure S2. Crude SWCNT separation by diameter. (a) Optical images of separated fractions diluted by a factor of 20 to reveal the colors of the suspensions: left (bottom phase), right (top phase). (b) Absorption spectra. 2D PLE maps of (c) top (T) and (d) bottom (B) phases.

The partitioning system contained 13.5 mL of DEX (70 kDa, 20%), 5.4 mL of PEG (6 kDa, 50%), 1.5 mL of DOC (5%), 1.5 mL of SWCNT dispersion (2% DOC; 1 mg/mL of SWCNTs), 18 mL of TX100 (2.5%), and 6 mL of H_2O, giving the total volume of 45.9 mL. This was a ten-fold increase of the values marked in Table S3 above, i.e. 1 350 µL of DEX (70 kDa, 20%), 540 µL of PEG (6 kDa, 50%), 150 µL of DOC (5%), 150 µL of SWCNT dispersion (2% DOC; 1 mg/mL of SWCNTs), 1 800 µL of TX100 (2.5%), and 600 µL of H_2O.

3. Partitioning Using SC SWCNT Dispersion and SC/TX100 Surfactant Mixture

Table S4. SC/TX-100/SC SWCNT sample series: composition and description. Each sample contained 1 350 µL of DEX (70 kDa, 20%), 540 µL of PEG (6 kDa, 50%), 360 µL of SC (10%), and 225 µL of SWCNT dispersion (2% SC; 1 mg/mL of SWCNTs). Total volume: 4 590 µL. of Selected samples (shaded rows) having intense colors are depicted in **Figure S3a**.

Sample number	2.5% TX-100 addition [µL]	DI water addition [µL]	Bottom phase color	Top phase color
1	0	2 115	Black	Colorless
2	50	2 065	Black	Colorless
3	100	2 015	Black	Gray/Green
4	150	1 965	Black/Purple	Green
5	200	1 915	Black/Purple	Green
6	250	1 865	Purple	Green
7	300	1 815	Purple	Green
8	350	1 765	Pink	Black/Green
9	400	1 715	Pink	Black
10	450	1 665	Pink/Colorless	Black
11	500	1 615	Pink/Colorless	Black
12	550	1 565	Faint blue	Black
13	600	1 515	Faint blue	Black
14	650	1 465	Faint blue	Black

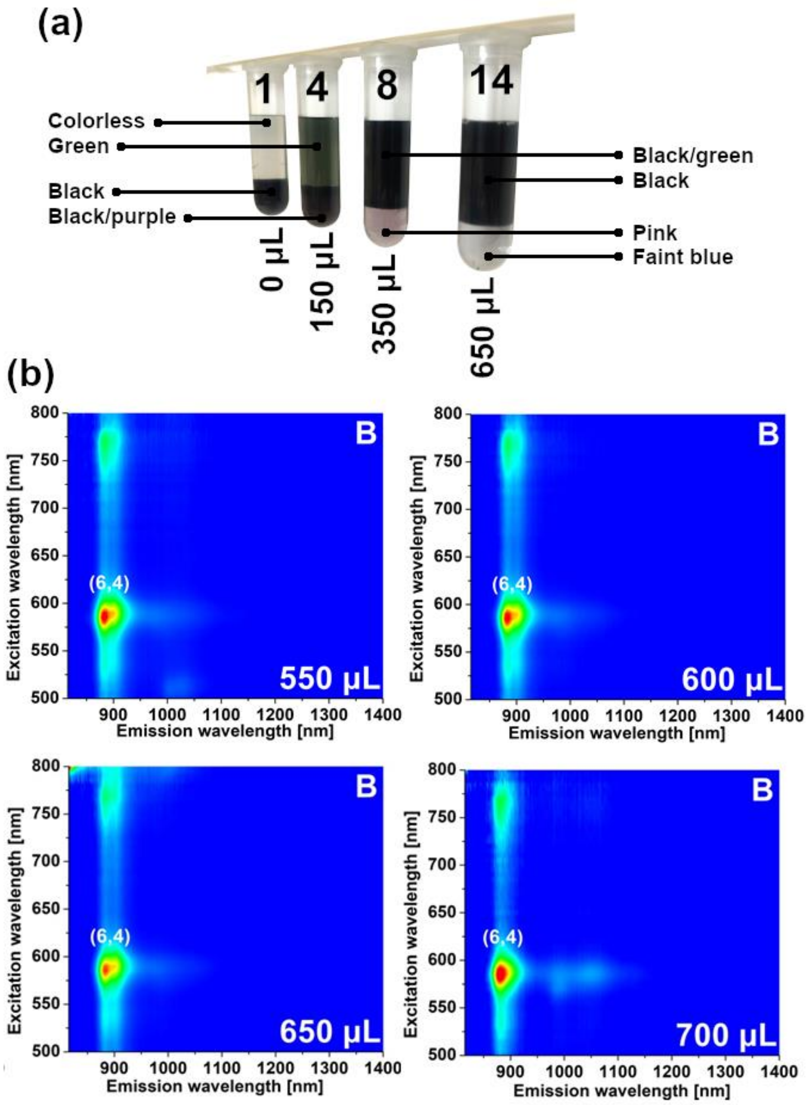

Figure S3. (a) Photographs of selected samples prepared according to conditions given in **Table S4** arranged in order of increasing 2.5% TX-100 volume, (b) PL excitation-emission maps of bottom samples depending on the TX100 addition volume (SC-dispersed SWCNTs were sorted).

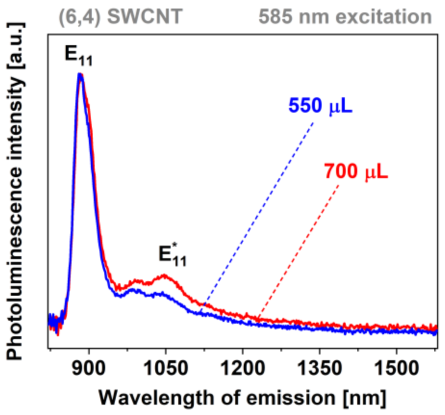

Figure S4. PL emission spectra from samples of (6,4) SWCNTs harvested by ATPE using 550 µL and 700 µL of 2.5% TX-100 solution.

4. Separation of a Spectrum of Different SWCNT Dispersions

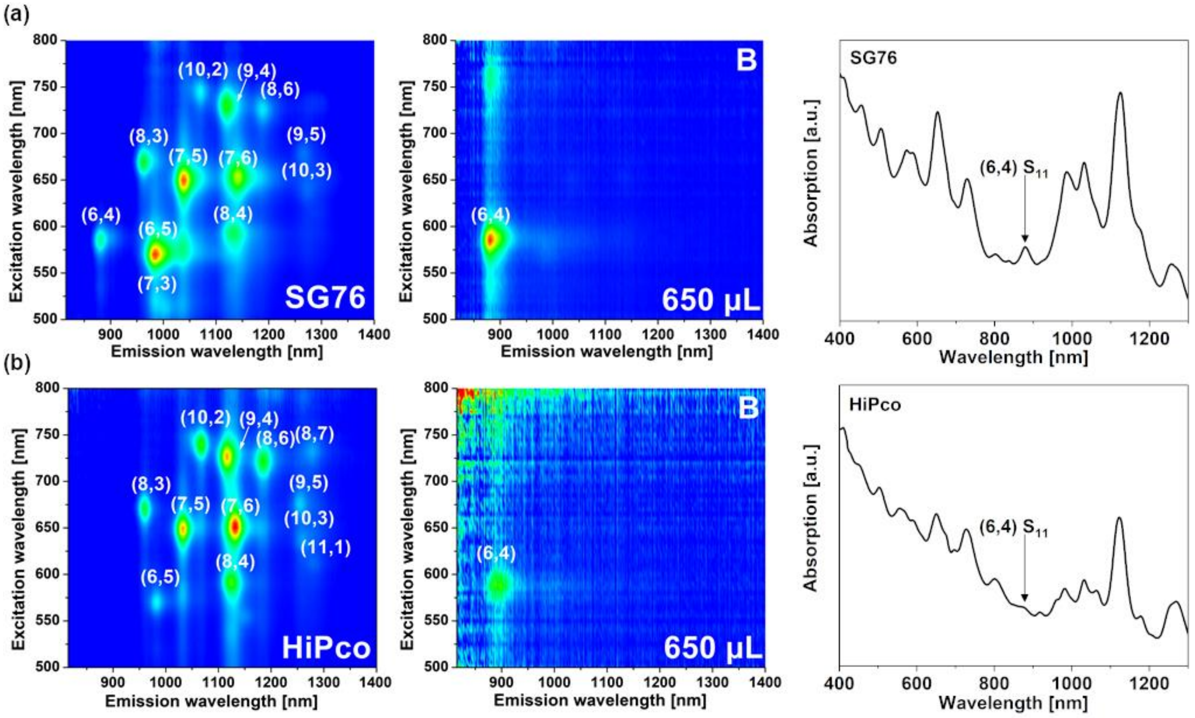

Figure S5. PL excitation-emission maps of starting materials (SG76 – top and HiPco – bottom) along with corresponding bottom samples for 650 µL TX100 addition volume. Corresponding absorption spectra of raw materials used for separation are provided, indicating that (6,4) SWCNTs are a minority fraction.

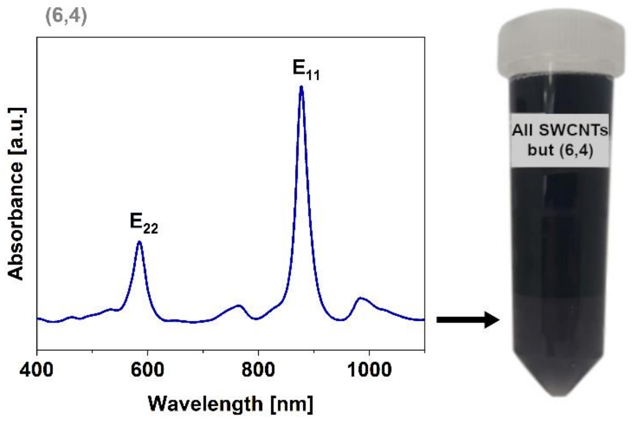

Figure S6. Absorption spectrum of (6,4) SWCNTs extracted from waste SWCNTs accumulated in our laboratory. The image shows SWCNTs partitioned between the top and bottom phases in the ATPE system. High-intensity color of the bottom phase indicates a high concentration of (6,4) SWCNTs therein. To prove the validity of the reported concept, the separation scale was increased ten-fold, which produced 45.9 mL of the ATPE system.

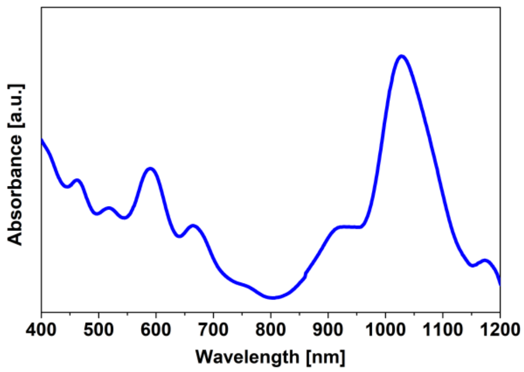

Figure S7. Absorption spectrum of TX100 SWCNT dispersion.

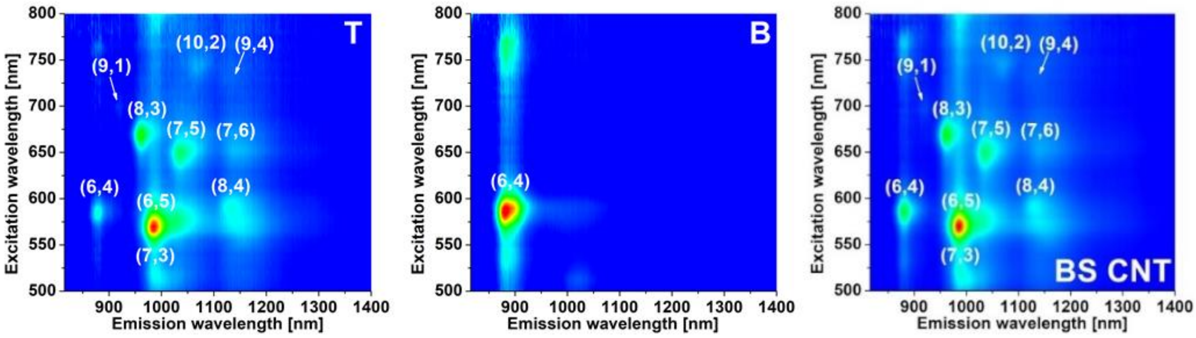

Figure S8. Results of experiments with binary surfactant SWCNT dispersion. PL excitation-emission maps of (T) top and (B) bottom phases. Starting binary surfactant (BS) SWCNT dispersion is shown on the right.

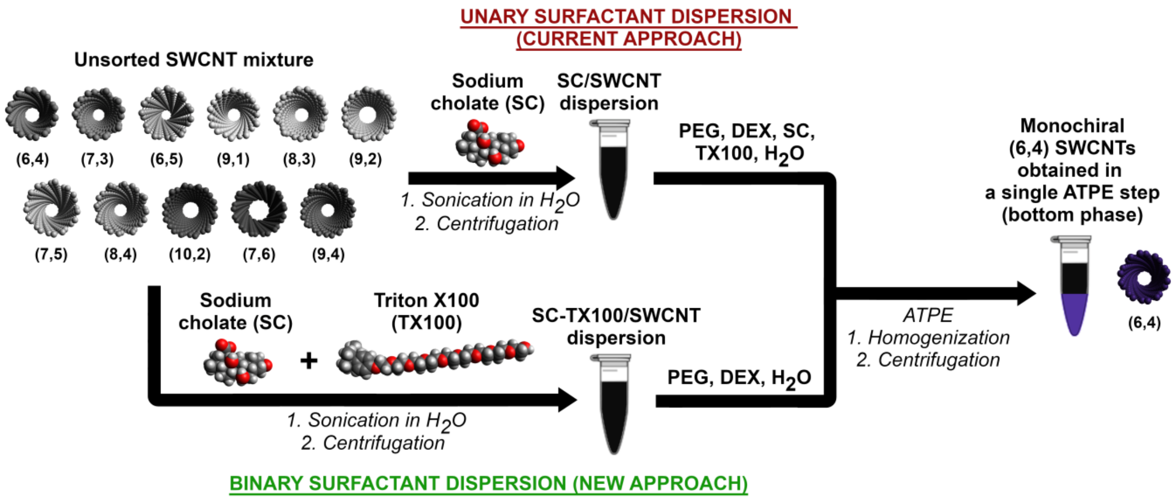

Figure S9. Comparison of typical and simplified ATPE approaches.

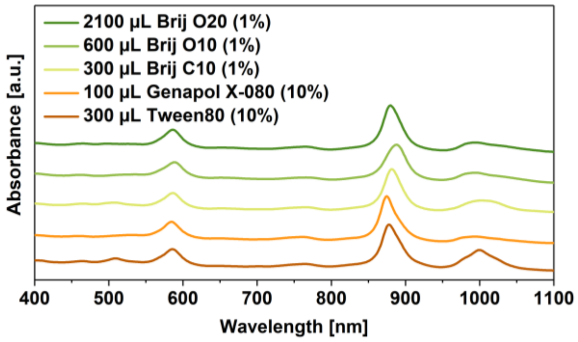

Figure S10. Bottom phase absorption spectra obtained with the use of various surfactants.

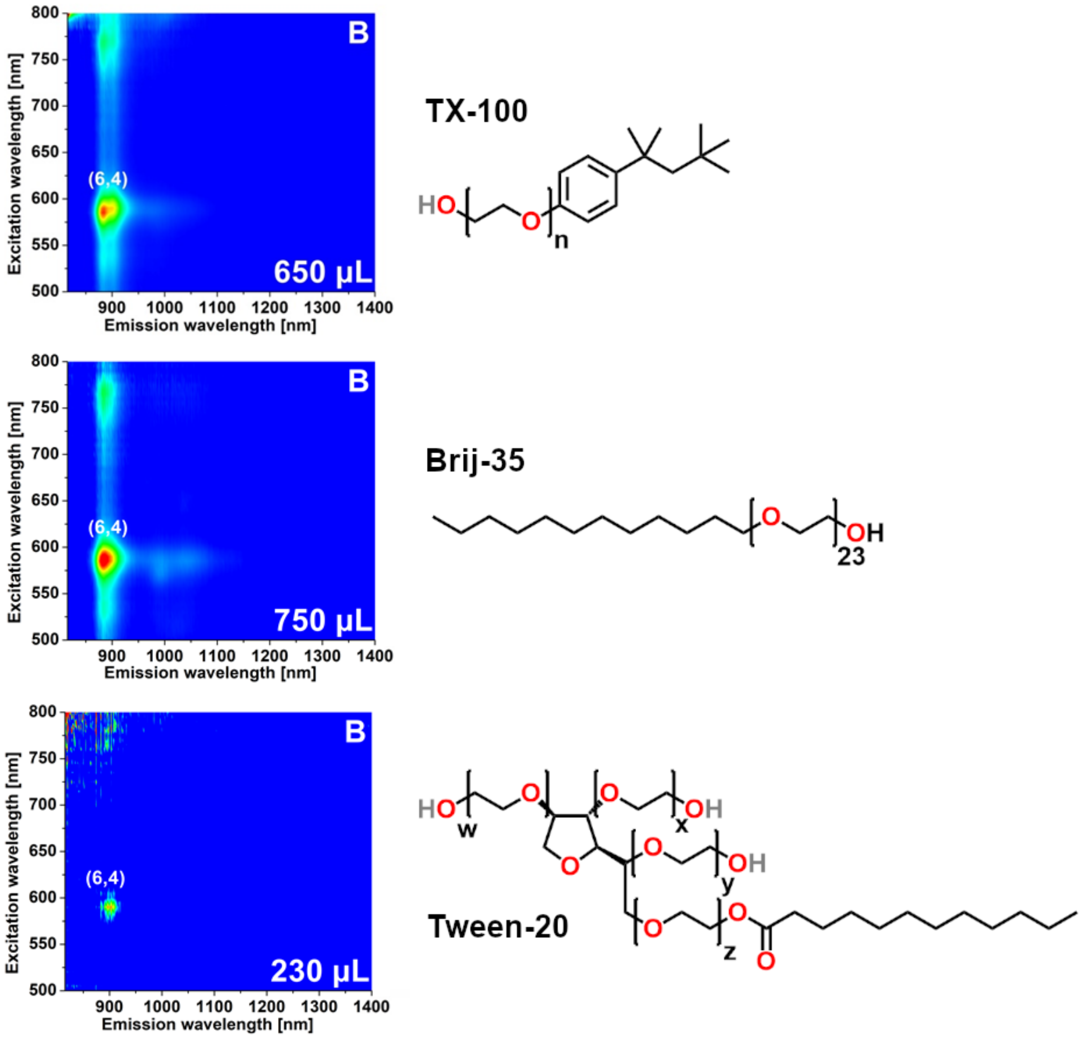

Figure S11. PL excitation-emission maps of bottom samples and structure of surfactants used to produce them. Inferior stability of SWCNT dispersions mediated by Tween-20 (which is a common problem of SWCNT dispersions made using non-ionic surfactants as visualized in Figure S7) may explain the lower quality of the PL map.

Table S5. SC/Brij-35/SC SWCNT sample series: composition and description. Each sample contained 1 350 µL of DEX (70 kDa, 20%), 540 µL of PEG (6 kDa, 50%), 360 µL of SC (10%), and 225 µL of SWCNT dispersion (2% SC; 1 mg/ml of SWCNT). Total volume: 4 590 µL.

5% Brij-35 addition [µL]	DI water addition [µL]	Bottom phase color	Top phase color
500	1 615	Pink	Black
705	1 415	Colorless	Black
720	1 395	Colorless	Black
735	1 380	Colorless	Black
750	1 365	Colorless	Black

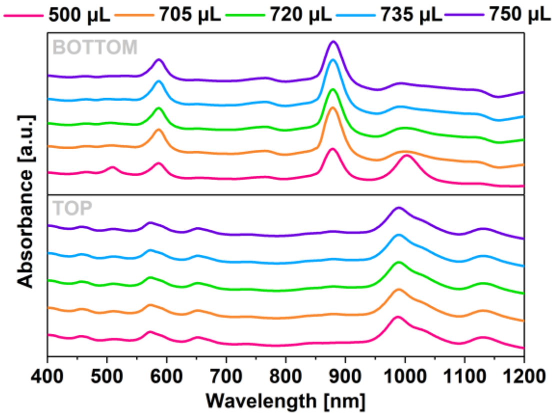

Figure S12. Absorption spectra of bottom and top fractions from SWCNTs separated using various amounts of Brij-35.

Table S6. SC/Tween20/SC SWCNT samples series: composition and description. Each sample contained 1 350 µL of DEX (70 kDa, 20%), 540 µL of PEG (6 kDa, 50%), 360 µL of SC (10%), and 225 µL of SWCNT dispersion (2% SC; 1 mg/ml of SWCNT). Total volume: 4 590 µL.

5% Tween-20 addition [µL]	DI water addition [µL]	Bottom phase color	Top phase color
50	2 065	Pink	Green
170	1 945	Colorless	Black
185	1 930	Colorless	Black
200	1 915	Colorless	Black
230	1 885	Colorless	Black

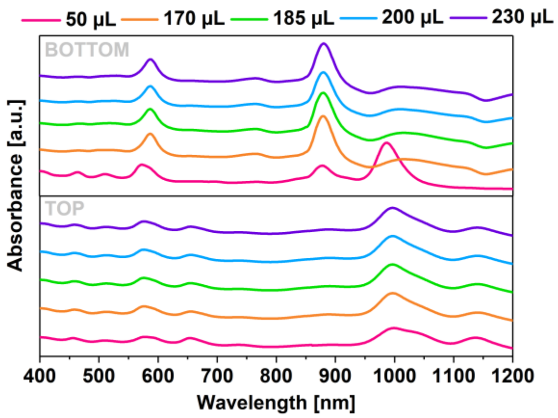

Figure S13. Absorption spectra of bottom and top fractions from SWCNTs separated using various amounts of Tween-20.

5. Elucidation of the Mechanism of the Separation

5.1. Calculations

Table S7. Characterization of surfactants. Please refer to the information given below for the methodology employed to determine the required content of non-ionic surfactants in "wt% necessary to extract (6,4) SWCNTs.

	TX100	Brij-35	Tween-20
**Molecular weight [g/mol]**	647	1 199.5	1 228
**CMC [mM][1]**	0.3	0.091	0.05
**Average micellar weight [kDa]**	48-106[2]	48[2]	37.1[3]
**Aggregation number [number]**	74-164	40	30
**HLB [-]**	13.5	16.9	16.7
**EO units [number]**	9-10	23	20
**EO units [%]**	61-68	84	72
**Structure**	Linear	Linear	Non-linear
**End group**	4-(1,1,3,3-tetramethylbutyl) phenyl	Lauryl	Dodecanoate
**Required amount to extract (6,4) SWCNT effectively [wt%]**	0.342	0.792	0.243

Below, we show the methodology used to determine the percentage of the specified non-ionic surfactants needed for reaching optimum (6,4) SWCNT isolation. First, we measured the densities of the stock solutions used for separating SWCNTs in the ATPE system through pycnometry (**Table S7**). Then, based on density measurements, we calculated the weights of each added solutions, via multiplication of the added volume (ml) by the established density values.

Table S8. Physicochemical parameters of stock solutions

Compound	Concentration of stock solution (w/w; water) [%]	Density [g/ml]	Volume [mL]	Weight [g]
DEX	20	1.078	1.350	1.456
PEG	50	1.093	0.540	0.590
SC	10	1.024	0.360	0.369
TX100	2.5	1.002	0.650	0.651
Brij35	5	1.005	0.750	0.754
Tween20	5	1.005	0.230	0.231
SWCNTs	2	1.006	0.225	0.226
Water	100	0.999	1.465	1.464
		TOTAL	4.590	4.756

The sample, in which 650 µL of the TX100 (2.5% aqueous solution) was employed, serves as an example of how to calculate the dry weight of non-ionic surfactant ($w_{TX100,dry}$) needed for extraction of (6,4) SWCNTs, which is obtained by calculating the product of weight of the solution by its concentration.

$$w_{TX100,dry} = w_{TX100,solution} \cdot c_{TX100,solution} = 651 \cdot 0.025 = 16.275\ mg$$

This value needs to be recalculated by considering the weight of the whole system (w_{ATPE_system}) to enable comparison between different surfactants.

$$wt\%\ of\ TX100 = \frac{w_{TX100,dry}}{w_{ATPE\_system}} \cdot 100\% = \frac{16.275}{4756} \cdot 100\% = 0.342\%$$

The weight % for Brij-35 (750 µL, 5%) and Tween-20 (230 µL, 5%) were determined analogously.

5.2. Simulations

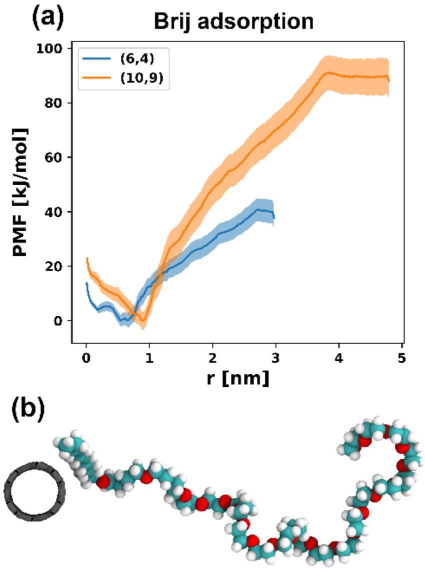

Figure S14. (a) Adsorption energy profiles for Brij-35 on (6,4) and (10,9) SWCNTs. (b) Visualization from the simulations demonstrating that longer separation distances are needed when performing US on long-chain surfactants – at a COM separation of 3 nm, the hydrophobic head is still adsorbed to the SWCNT surface.

5.2.1. Calculation of Surfactant/SWCNT Distances

Each surfactant molecule was labeled using a vector connecting two characteristic atoms. The vector definition for the two surfactant molecules is shown below in **Figure S15**.

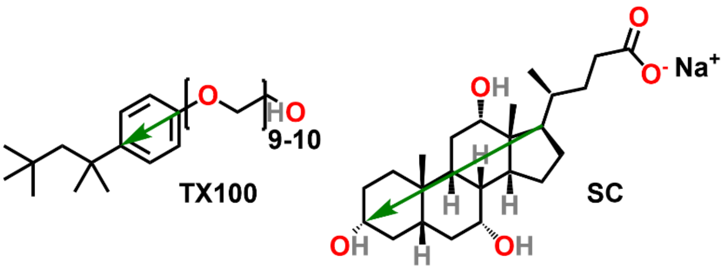

Figure S15. Definition of molecular vectors used for distance and orientation calculations.

The distance in the XY-plane between the SWCNT axis and the center point of the molecular vector is calculated as a function of time for each individual surfactant molecule. Each trace in the **Figures 7a, 7b, and S16**, therefore, represents a single surfactant molecule. When a trace transitions from a high value to a low value, this is indicative of an adsorption event.

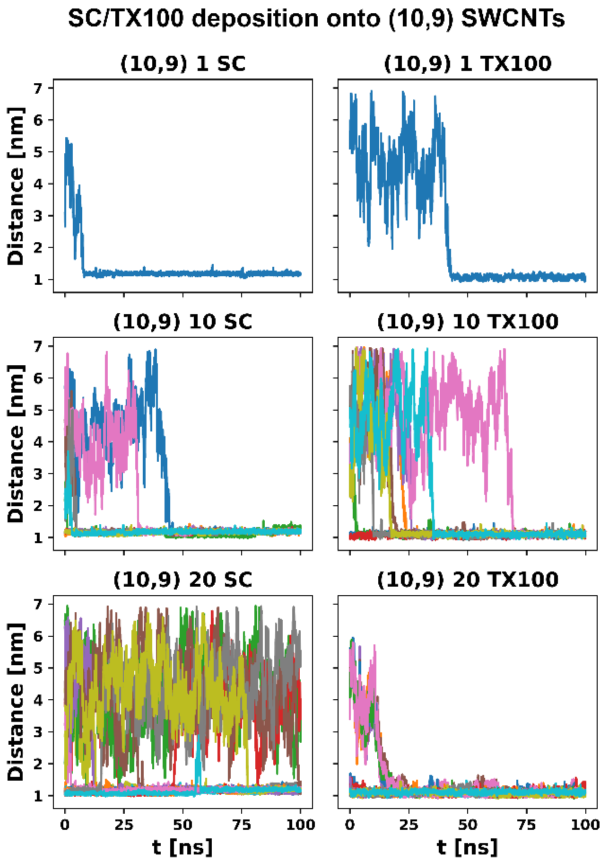

Figure S16. Individual surfactant distances as a function of the number of deposited surfactant molecules (SC or TX100) and time for (10,9) SWCNTs.

Overall, the results (**Figure 7** and **S16**) show that the kinetics of corona formation is more rapid in the case of TX100 deposition of SWCNT surface (compared to SC). This is most evident in the simulations, wherein 20 surfactant molecules are considered. Minor deviations in the case of systems including a smaller number of surfactant molecules stem from the statistical uncertainty of these discrete systems (only 1 or 10 molecules present as opposed to the much larger number in the experiments).

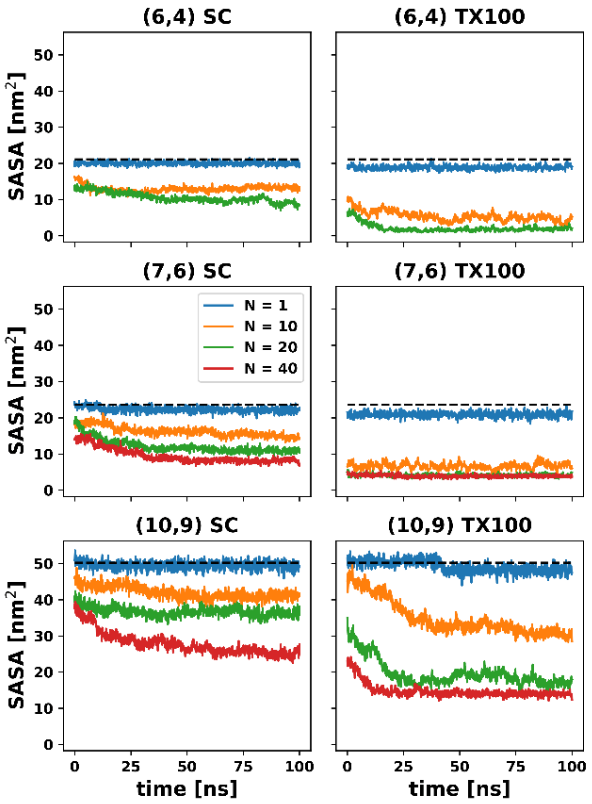

Figure S17. Absolute SASA values for the (6,4), (7,6), and (10,9) SWCNTs suspended using N=1, 10, 20, and 40 molecules of (left) SC and (right) TX100. The dashed line denotes the SASA of a bare SWCNT.

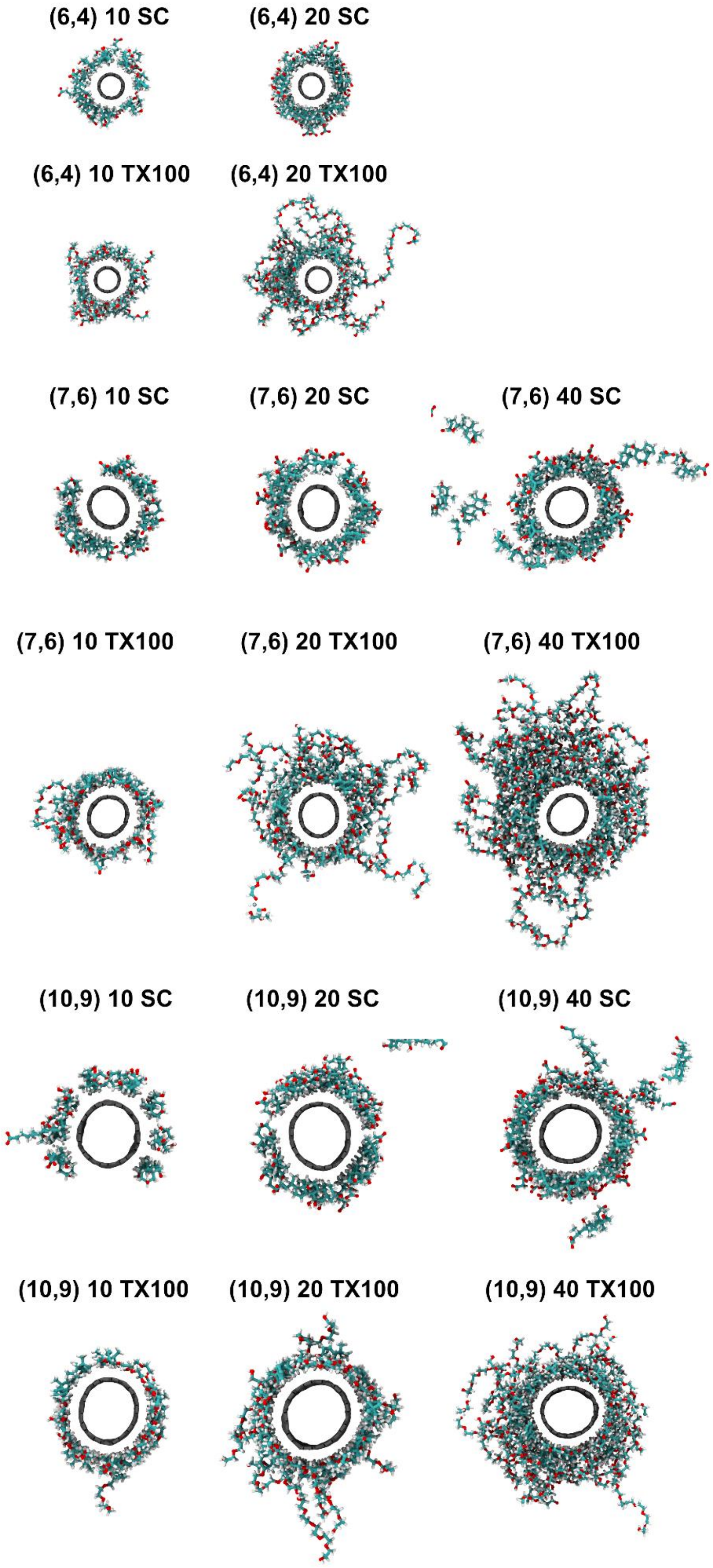

Figure S18. Snapshot of the surfactant/SWCNT complex for each system.

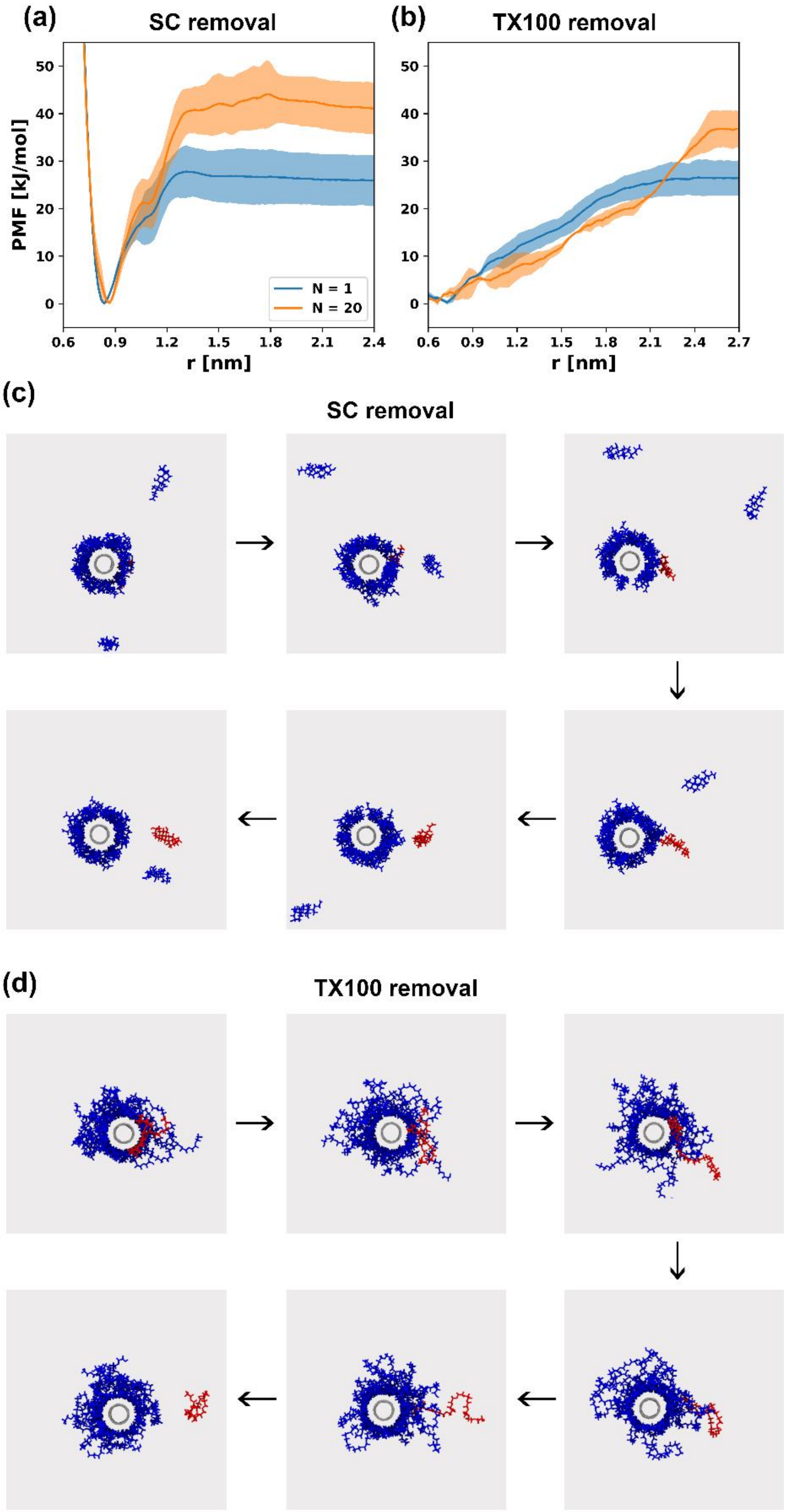

Figure S19. Comparison of PMFs for single surfactant (N=1) molecule removal from bare (6,4) SWCNT and from a pre-assembled corona consisting of 20 surfactant molecules (N=20): (a) SC, and (b) TX100 removal. Each curve averages 3 independent US trials and the uncertainty represents one standard deviation. Representative snapshots for the (c) SC and (d) TX100 systems with SWCNTs.

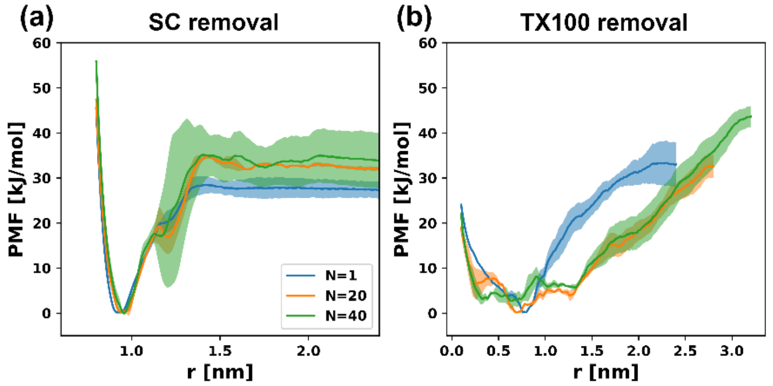

Figure S20. PMFs to remove a single surfactant molecule from a corona consisting of N=1, 20, and 40 molecules of (a) SC and (b) TX100 deposited on (7,6) SWCNTs.

5.2.2. Corona Structure

The overall goal is to understand how the surfactant molecules arrange themselves on the surface and to detect if this changes with respect to the SWCNT chirality or the surfactant concentration. Radial distribution functions (RDFs) are used to analyze the configuration of preferred molecular adsorption by quantifying the distance between the SWCNT axis and various atoms within the surfactant molecule. This analysis results in a cylindrical density profile of these specific atoms around the tube axis. **Figure S21** defines which atoms (named and labeled with grey circles) in the various molecules are used for RDF calculation.

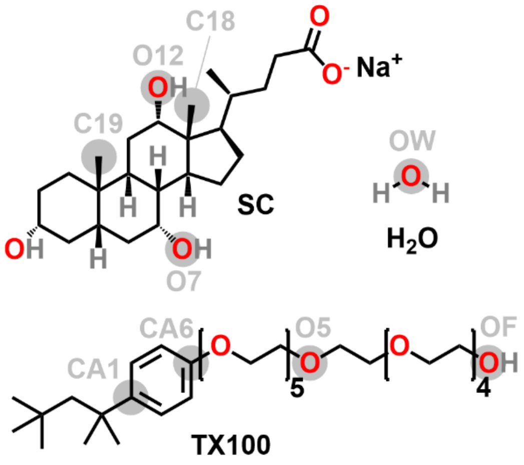

Figure S21. Atom labeling used during the determination of RDFs.

Since water is present in all systems investigated, we first examine its density around the SWCNT (**Figure S22**). The main observation is that as more TX100 is added, the water is increasingly displaced from the surface due to increased coverage by the larger TX100 molecules. As previously observed, the SC corona formation covers less of the surface as the cholate molecules are much smaller, therefore, water is still able to reach the SWCNT surface.

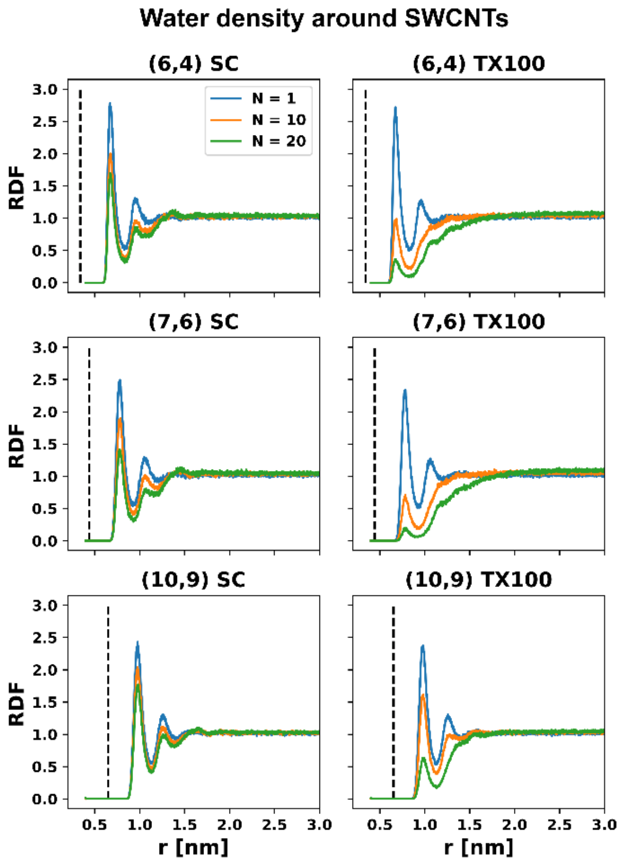

Figure S22. RDF of water molecules (N=1, 10, or 20) around SWCNT for each system. The dashed line represents the center of the carbon atoms forming the SWCNT.

Because the cholate is a more complex molecule, it is necessary to examine several atomic RDFs to understand its orientation with respect to the SWCNT surface. For the case where there is only a single cholate molecule, each of the C18, C19, and O12 atoms (top rows of **Figure S23** and **Figure S24**) seem to be equidistant from the SWCNT surface while the O7 atom is further away. This suggests that the cholate molecules prefer to adsorb in a configuration that is somewhat “on edge” as depicted in **Figure S25**. As more cholate molecules are added, this preferred orientation is disrupted, and several other configurations are adopted, as evidenced by the appearance of multiple peaks in the RDFs. However, in general, it appears that the O7 atom prefers to remain the furthest from the surface of the 4 observed atoms. Ultimately, it seems that neither the chirality nor diameter of the SWCNT does not affect the RDFs of the SC molecule. All changes are due to the increased number of cholate molecules during corona formation.

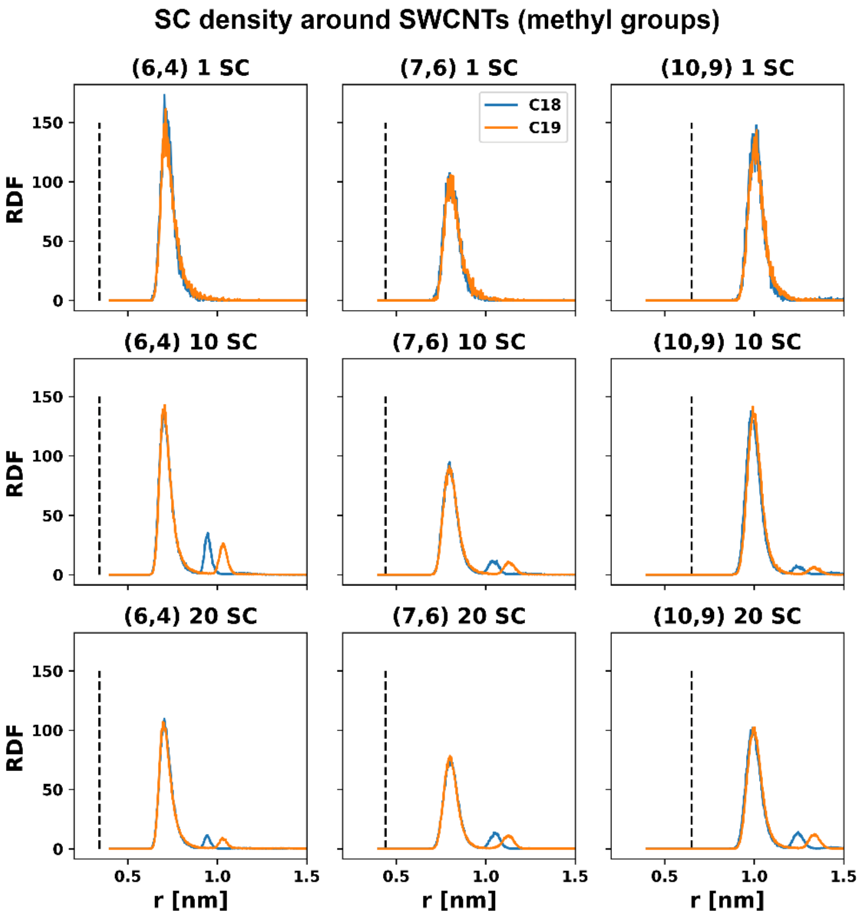

Figure S23. RDF of SC methyl groups (C18 and C19) around SWCNT for each system. The dashed line represents the center of the carbon atoms forming the SWCNT.

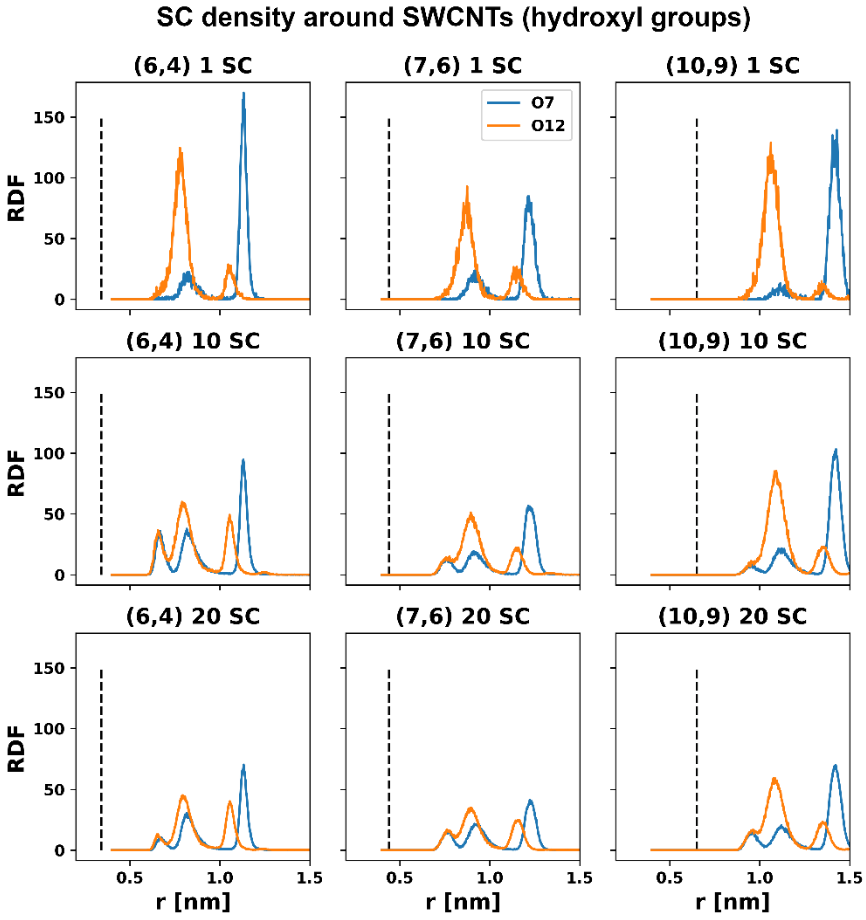

Figure S24. RDF of SC hydroxyl groups (O7 and O12) around SWCNT for each system. The dashed line represents the center of the carbon atoms forming the SWCNT.

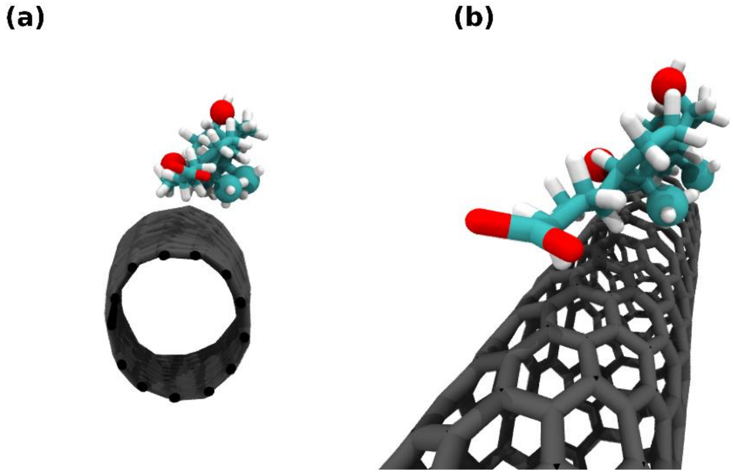

Figure S25. Two views of the same system demonstrating the preferred "on-edge" configuration of an adsorbed cholate molecule. Notice the C18 and C19 atoms (cyan spheres), and the O12 atom (red sphere closest to SWCNT surface) are roughly equidistant from the surface.

Since TX100 is a linear, chain-like molecule, it is important to examine RDFs of both the aromatic head group and the tail to determine its general adsorbed conformation. The RDFs of the aromatic carbons (**Figure S26**) seem to suggest that both SWCNT diameter and surfactant concentration play a role in the adsorbed configuration, especially when considering the (10,9) SWCNT. This could potentially be due to pi-stacking between the aromatic ring in TX100 and the aromatic graphene lattice. The RDF of the flexible tails does not display these dependencies (**Figure S27**), but they do indicate that the tails are also adsorbed onto the surface (although much less so in the case of 20 TX100 molecules). Ultimately, these results suggest that much greater conformational space is sampled by the adsorbed TX100 molecules compared to SC which is rather limited in how it adsorbs onto the surface.

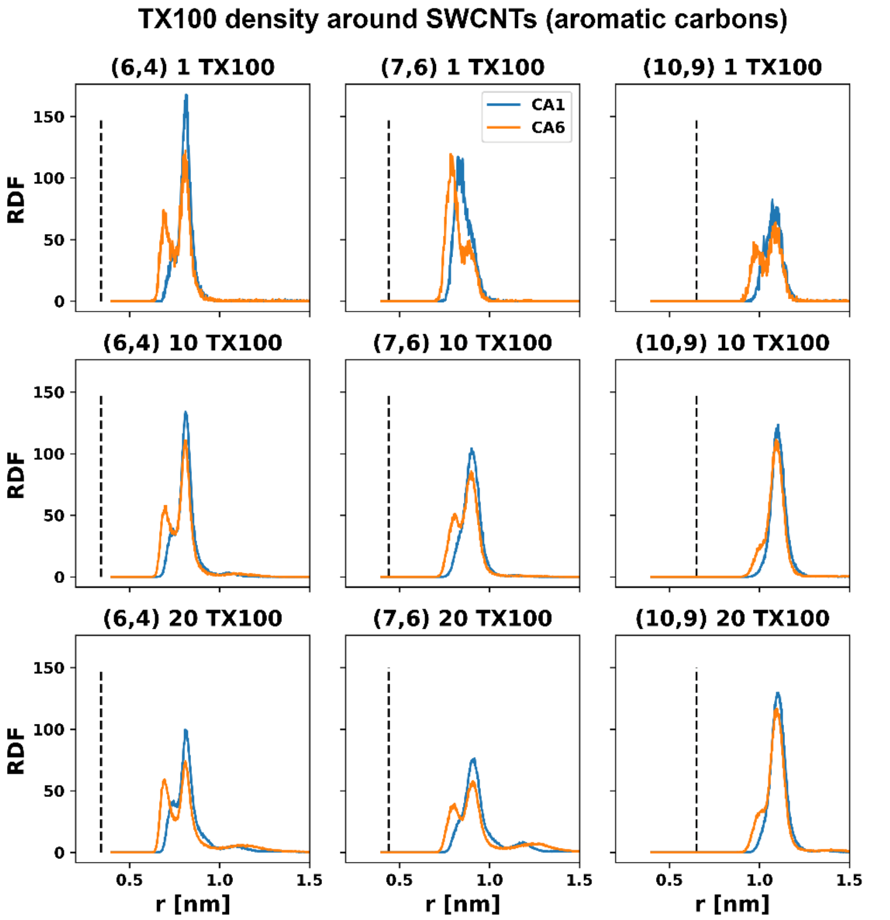

Figure S26. RDF of TX100 aromatic carbons (CA1 and CA6) around SWCNT for each system. Dashed line represents the center of the carbon atoms forming the SWCNT.

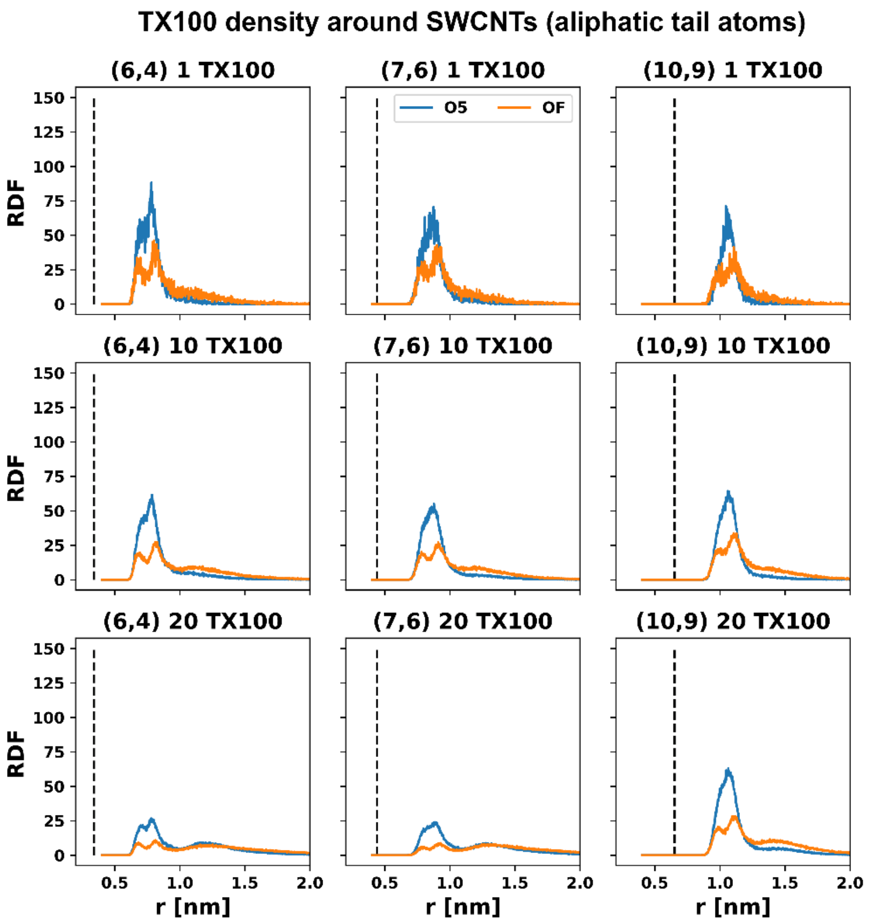

Figure S27. RDF of TX100 aliphatic tail atoms (O5 and OF) around SWCNT for each system. The dashed line represents the center of the carbon atoms forming the SWCNT.

Finally, we quantify the orientation of the adsorbed surfactants with respect to the SWCNT by measuring the dihedral angle between the surfactant vector defined above in **Figure S15** and a vector that lies on the z-axis of the SWCNT (see **Figure S28** for examples pertaining to cholate). This approach is useful as it helps describe the angular relationship between the adsorbed molecule and the SWCNT – a quantity that is not captured via simple RDFs. This orientation was measured for each surfactant molecule as a function of time, and a histogram was created of all the dihedral angles observed during the simulation (**Figure S29**).

At first glance, it appears that most surfactants prefer to adsorb in configurations that align their molecular vector with the tube axis (peaks at 0° and 180°). However, there are several instances where this is not the case. For example, as the number of cholate molecules increases on the (6,4) SWCNT, there is an increase in the number of molecules that assume angles perpendicular to the SWCNT axis (±90°). Another is the large peak at -90° for a single TX100 on the (7,6) SWCNT. This distribution of dihedral angles is the quantity that seems to be most affected by surfactant identity, surfactant concentration, and SWCNT diameter. It correlates with the packing of different surfactant molecules during corona formation.

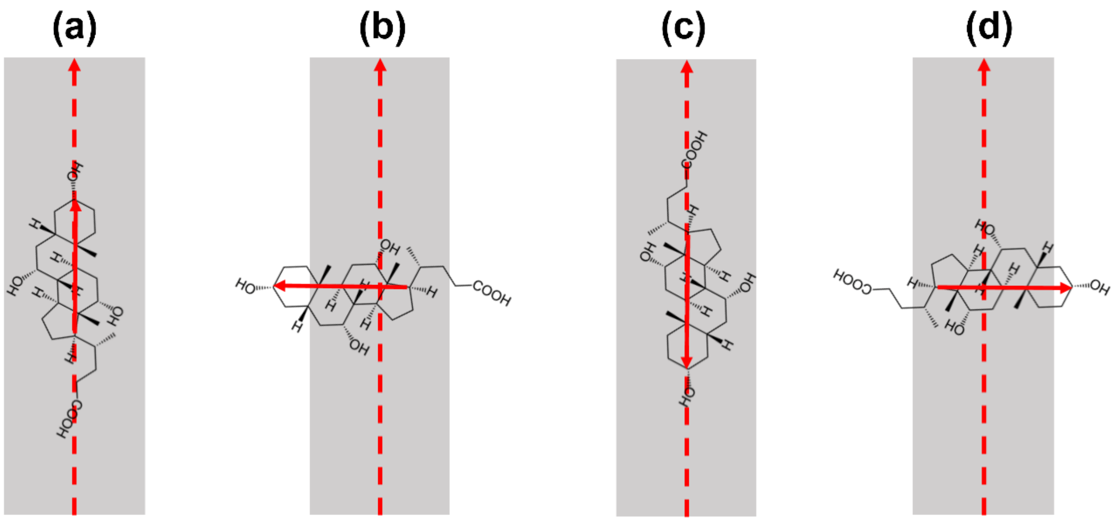

Figure S28. Demonstration of various dihedral orientations of SC molecules with respect to the SWCNT (in grey). (a) 0° (b) -90° (c) ±180° (d) 90°. The aromatic head of the TX100 molecules can also arrange themselves analogously.

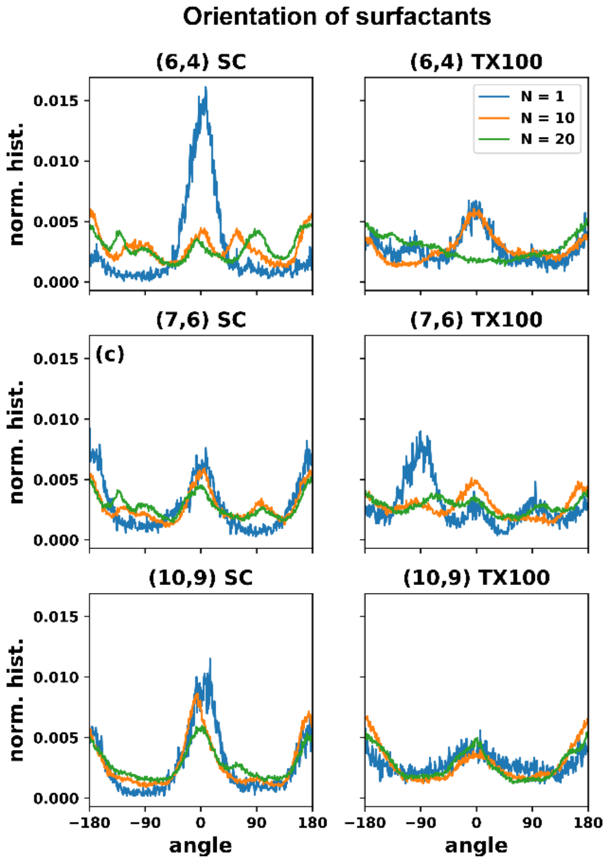

Figure S29. Histograms of the dihedral angle between the molecular vector of the surfactant molecules (N=1, 10, or 20) and the z-axis of the SWCNT.

5.3. Mechanism of the Partitioning Process

Recent results[4] provide additional evidence explaining why (6,4) SWCNTs prefer to persist in the bottom phase even at relatively high concentrations of non-ionic surfactants. According to the referred study, (6,4) SWCNTs are tightly fitted by 7 sodium deoxycholate molecules around the circumference. The situation may be similar in the case of SC-dispersed SWCNTs since the structures of these two surfactants are very similar. In this case, it is reasonable to conclude that replacing SC on the surface with TX100 should be challenging.

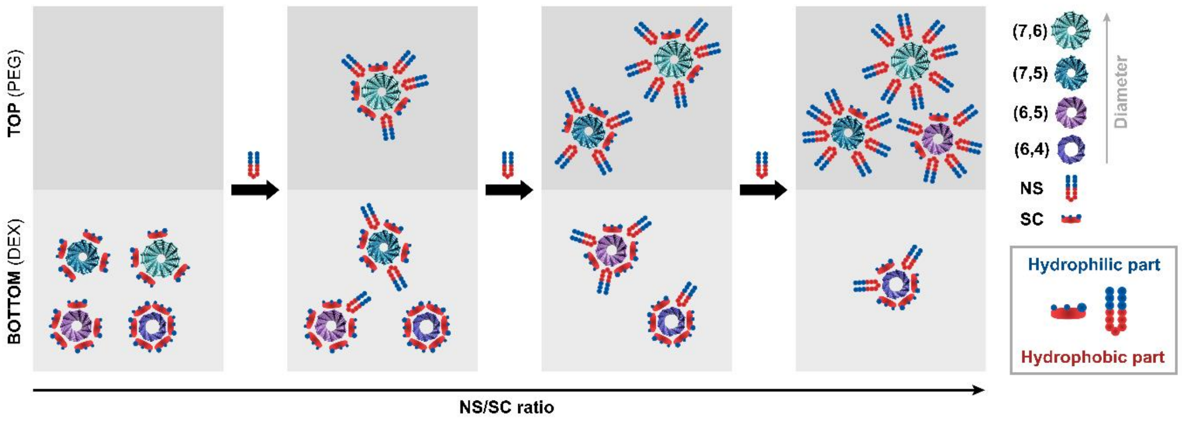

Figure S30. Illustration of the favored migration of large-diameter SWCNTs to the top phase caused by addition of non-ionic surfactant molecules, which replace SC on the surface of SWCNTs.

Besides (6,4), other chiralities can also be isolated in analogously but not in a single step because the employed surfactant system differentiates SWCNTs by diameter. In such a case, one needs to collect a bottom- or top-phase sample of the SWCNTs of interest and combine it with a corresponding fresh top- or bottom-phase solution to recreate the biphasic system. Then solutions of bile salt surfactants or non-ionic surfactants can be engaged to promote the shift of SWCNTs to the bottom- or top-phase, respectively. Typically, two such components are necessary to enable the competitive adsorption of surfactants, which is at the heart of the ATPE approach[5]. A synergistic effect was obtained through the simultaneous use of anionic and non-ionic surfactants - adequate SWCNT debundling and diameter-specific binding to selected SWCNT types were promoted, respectively.

6. References

[1] X. Zeng, J. Quaye, K. Osseo-Asare, *Colloids and Surfaces A: Physicochemical and Engineering Aspects* **2004**, *246*, 135.

[2] T. Arnold, D. Linke, *Current Protocols in Protein Science* **2008**, *53*, 1.

[3] C. Carnero Ruiz, J. Molina-Bolívar, J. Aguiar, G. MacIsaac, S. Moroze, R. Palepu, *Colloid and Polymer Science* **2003**, *281*, 531.

[4] J. Defillet, M. Avramenko, M. Martinati, M. Á. López Carrillo, D. Van der Elst, W. Wenseleers, S. Cambré, *Carbon* **2022**, *195*, 349.

[5] H. Li, G. Gordeev, O. Garrity, N. A. Peyyety, P. B. Selvasundaram, S. Dehm, R. Krupke, S. Cambré, W. Wenseleers, S. Reich, M. Zheng, J. A. Fagan, B. S. Flavel, *ACS Nano* **2020**, *14*, 948.
